# Understanding non-linear effects from Hill-type dynamics with application to decoding of p53 signaling

**DOI:** 10.1038/s41598-018-20466-2

**Published:** 2018-02-01

**Authors:** Xiaomin Shi, Jeffrey R. Reimers

**Affiliations:** 1 International Centre for Quantum and Molecular Structures and Mathematics Department, Shanghai University, Shanghai, 200444 China; 2 International Centre for Quantum and Molecular Structures and Physics Department, Shanghai University, Shanghai, 200444 China; 3 School of Mathematical and Physical Sciences, University of Technology Sydney, Sydney, NSW 2006 Australia

## Abstract

Analytical equations are derived depicting four possible scenarios resulting from pulsed signaling of a system subject to Hill-type dynamics. Pulsed Hill-type dynamics involves the binding of multiple signal molecules to a receptor and occurs e.g., when transcription factor p53 orchestrates cancer prevention, during calcium signaling, and during circadian rhythms. The scenarios involve: (i) enhancement of high-affinity binders compared to low-affinity ones, (ii) slowing reactions involving high-affinity binders, (iii) transfer of the clocking of low-affinity binders from the signal molecule to the products, and (iv) a unique clocking process that produces incremental increases in the activity of high-affinity binders with each signal pulse. In principle, these mostly non-linear effects could control cellular outcomes. An applications to p53 signaling is developed, with binding to most gene promoters identified as category (iii) responses. However, currently unexplained enhancement of high-affinity promoters such as CDKN1a (p21) by pulsed signaling could be an example of (i). In general, provision for all possible scenarios is required in the design of mathematical models incorporating pulsed Hill-type signaling as some aspect.

## Introduction

Signaling dynamics involving networks is now recognized as an important way in which biochemical information is encoded^1–6^. It has many applications including: the role of transcription factor p53 in cancer prevention^4,7–10^, calcium signaling^11–16^, circadian rhythms^17^, yeast stress-response transcription factor Msn2^18^, endothelial growth arrest^19^, signal related kinase^20^, transcription factor NF-κB^21^, transcription factor GtaC^22,23^, and more^1–5^. In all cases, signals are decoded to generate multiple cellular responses and outcomes^12,18,24–27^, presenting pharmacological targets^9,10,27–30^.

Many signaling scenarios involve Hill-type dynamics involving binding of multiple signal molecules (S) to a receptor (R)^31–36^, and in many situations the concentration of the signal molecule oscillates with time. We present numerical and analytical solutions to the simplest model^8,15,18^ of Hill-type dynamics under the influence of pulse signaling. How such a model can be applied to any specific biochemical problem requires careful consideration^34^. Nevertheless, various starkly different basic scenarios emerge, scenarios depicting very different connections between signaling variations and system outcome.

**Figure 1:**
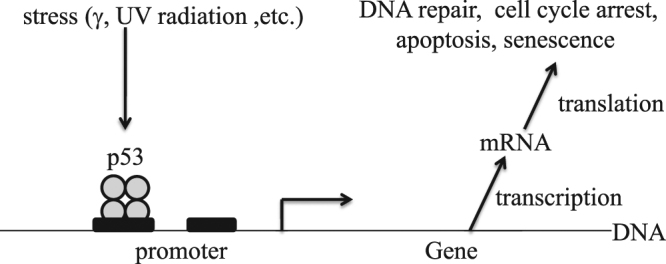
Model for how p53 dynamics drives gene expression. Four p53 molecules bind to the DNA promoter that regulates gene expressions, including transcription to make mRNA. Downstream processes then induce translation to influence cellular outcomes.

One application of the developed analytical and numerical solutions is presented, focusing on how p53 signaling is decoded. Figure 1 sketches the processes associated with p53 binding to promoters of various genes, triggering gene transcription and expression. This system is important as half of human cancers are associated with p53 mutations while the remainder show anomalies with p53 signaling instabilities^7^. Like cancer itself^6^, processes involving p53 are extremely complex^10,27,37^ as p53 activates hundreds of genes^10,30^ as well as participating in many processes not involving gene expression^38^. Temporal changes in the concentration of active forms of p53 are utilized to suppress tumor growth and to prevent the propagation of damaged cells^39,40^. Changes may be simply to the concentration of p53, involve generation of a single transient p53 pulse, or involve the generation of a p53 pulse sequence^1,41–43^. Modern research tends to focus on this *complexity* and its detailed interpretation and/or modelling. From a biological perspective, recent work is therefore focusing on finding emergent simpler pictures, considering e.g., high-throughput studies of the effects of p53 on thousands of genes^44^ and effects preserved over cell lines^37^. Although the encoding of p53 pulses has been the subject of much research and is well understood^4,8–10,41,45–57^, much remains unknown concerning complex problems like how these signals are decoded by gene promoters and how expressed proteins lead to biochemical function.

Computational modeling provides a popular means by which the complexity of p53 signaling can be addressed; in principle, it is possible simply to keep adding reactions until all important chemical features are included in the model. While early mathematical models for p53 pulsed signal generation and/or its decoding were relatively simple^41,45–49,58–60^, in recent years, many intricate biochemical models have been developed^4,8–10,50–57^ focusing on the complexity of p53 interactions. However, *four* molecules of p53 are required to bind to gene promoters^45,61–63^, opening up the possibility of Hill-type mechanisms that need be discussed in terms of pulsed signaling. Currently there is great variation amongst models as to if and how Hill-type signaling is included. Sometimes it is fully included^8,37,48,52–54,56,58–60^, sometimes it is partially included in only the cycle with WIP1 and Mdm2^9^, sometimes partially included^57^ through use of equations derived assuming that pulsed signaling is inconsequential, and sometimes it is omitted altogether^49–51,55^. In any case, recent models are so complex and contain so many arbitrarily determined parameters that effects coming from the multiple binding of p53 to promoters are difficult to identify, with summaries of many such models not registering intrinsic features of pulsed Hill-type mechanisms as being significant in the big picture^10,37,57,64^.

Binding affinities are known for p53 with some key gene promoters, and it is known that predictions of steady-state promoter binding based upon them do not correlate with observed downstream function^7,65,66^. We consider whether or not signal pulsing could enable a correlation to be found, focusing on as yet uninterrupted results^4^ showing how pulsed signaling can enhance expression of certain genes compared to others.

In this work we derive many analytical results depicting pulsed Hill-type signaling. An approach allowing application to p53 signal decoding is then developed and applied, focusing on explaining observed pulsing effects. The scenarios presented for pulsed Hill-type signaling are expected to be widely applicable to many pulsed signaling scenarios.

## Results

### Square-wave pulsed Hill-type signaling

**Figure 2:**
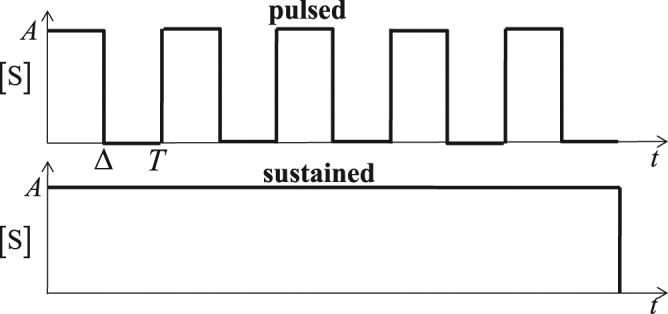
Square-wave pulsed signaling model depicting the temporal variation of the signal-molecule concentration [S] where *A* is the pulse amplitude, Δ the pulse-on duration, *T* the pulsing period, and γ = Δ/T is the duty cycle.

We assume that pulsing of signal molecule S takes the form of a square wave as described in Fig. 2. This wave has a period *T*, signal-on duration time Δ, and duty cycle γ = Δ/*T* expressed as a function of time *t* as

1 $$[{\rm{S}}]=\{\begin{array}{cc}A & (i-1)T\le t < (i-1)T+{\rm{\Delta }}\\ 0 & (i-1)T+{\rm{\Delta }}\le t < iT\end{array}\quad i=1,2,.....,$$

Also considered is a sustained signaling scenario in which a single pulse occurs. In each case, the maximum concentration of the signal molecule is taken to be [S]_max_ = *A*. We also assume that the receptor R is of very low concentration compared to that of the signal molecule, as would be the case always for binding to gene promoters. The Hill coefficient^32,35^ *n* represents the number of molecules of S that must bind to R to activate the intended process (e.g., gene transcription):

2 $$n{\rm{S}}+{\rm{R}}\underset{{k}_{2}}{\overset{{k}_{1}}{\rightleftarrows }}{\rm{R}}{{\rm{S}}}_{n}$$

where *k*_1_ and *k*_2_ are the rate constants for association and disassociation, respectively, so that the dissociation equilibrium constant *K*_*d*_ and dissociation constant *K*_*A*_ are given by^32^

3 $${K}_{d}={K}_{A}^{n}={k}_{2}/{k}_{1}.$$

The rate of change of concentration of bound species is then given by standard chemical kinetics^36^ as

4 $$\frac{d}{dt}[R{S}_{n}]={k}_{1}[R]{[S]}^{n}-{k}_{2}[R{S}_{n}]$$

which, by introducing the binding probability *P*(*t*) as the ratio of [*RS*_*n*_] to the total receptor concentration [*R*]+[*RS*_*n*_], can be rewritten simply as^35^

5 $$\frac{dP(t)}{dt}=(1-P(t)){k}_{1}{[{\rm{S}}]}^{n}-{k}_{2}P(t).$$

Assuming [S] ≫ [R], we take the time dependence of the signal-molecule concentration [S] to be simply that provided by the external signal encoding chemistry sketched in Fig. 2.

### Analytical solution

Some aspects of square-wave pulsed Hill signaling have been solved analytically in the context of Msn2 signaling^18^, while extended equations involving also downstream reactions have been solved analytically^15^ for pulsed calcium signaling^11,15,33^, with many general principles revealed. Here we adapt and extend these analyses to focus on the binding of the signal molecule itself, simplifying it to its essentials. More details of the analytical solution for *P*(*t*) as a function of *k*_1_, *k*_2_, *A*, γ, and *T* are given in SI, along with derivations of analytical results in key limits. The solution is presented introducing ξ_*i*_ = *t* − (*i* − 1)*T* as the time elapsed since the beginning of the *i*-th pulse. The binding change during this pulse can be expressed as a rising component appropriate for the time interval 0 ≤ ξ_*i*_ ≤ Δ during which the signal-molecule concentration is high,

6 $$\frac{{P}_{i}({\xi }_{i})}{{\bar{P}}_{sus}}=1-\frac{{e}^{-{\xi }_{i}({k}_{1}{A}^{n}+{k}_{2})}}{1-{e}^{-({k}_{1}{A}^{n}{\rm{\Delta }}+{k}_{2}T)}}[(1-{e}^{{k}_{2}{\rm{\Delta }}-{k}_{2}T})+{e}^{-i({k}_{1}{A}^{n}{\rm{\Delta }}+{k}_{2}T)}({e}^{({k}_{1}{A}^{n}+{k}_{2}){\rm{\Delta }}}-1)]$$

and a falling component for the time interval Δ ≤ ξ_*i*_ < *T* during which the signal-molecule concentration is low,

7 $$\frac{{P}_{i}({\xi }_{i})}{{\bar{P}}_{sus}}=\frac{({e}^{{k}_{2}{\rm{\Delta }}}-{e}^{-{k}_{1}{A}^{n}{\rm{\Delta }}})(1-{e}^{-i({k}_{1}{A}^{n}{\rm{\Delta }}+{k}_{2}T)})}{1-{e}^{-({k}_{1}{A}^{n}{\rm{\Delta }}+{k}_{2}T)}}{e}^{-{k}_{2}{\xi }_{i}},$$

where

8 $${\bar{P}}_{sus}=\frac{{A}^{n}}{{K}_{A}^{n}+{A}^{n}}$$

gives the binding probability for sustained signaling at infinite time. Analytical solutions have also been obtained for a Hill model subject to sinusoidal signaling with implicit treatment of downstream kinetics included^8^, but these are very complex and have not been shown to lead to simple understanding.

### Characteristic numerical results

Quantitative application of the square-wave Hill-type pulsed-signaling model requires knowledge of the chemical parameters *n*, *k*_1_, and *k*_2_ for the binding of S to R, the peak S concentration *A*, the duty cycle γ and the clocking period *T*. However, instead of treating *k*_2_ as an independent variable, we depict results as a function of *K*_*A*_ = (*k*_2_/*k*_1_)^1/*n*^ and *k*_1_. This approach is useful if only *K*_*A*_ is known rather than both *k*_1_ and *k*_2_ as the diffusion limit often controls *k*_1_, (e.g., as is believed for p53^67^), making it is somewhat insensitive to variations, while *k*_2_ is controlled by molecular properties and environment^68,69^ and can vary dramatically. Typically values for γ and *T* would be known as these are relatively easy to measure experimentally, while experiments measuring *A* could be envisaged. Under these conditions, the binding probability as a function of time and dissociation constant *P*(*t*;*K*_*d*_) could be modeled considering feasible limits for *k*_1_ and appropriate values of *n*, *T*, *A*, and γ. Seeking general scenarios for pulsed Hill-type signaling, we take this approach.

**Figure 3:**
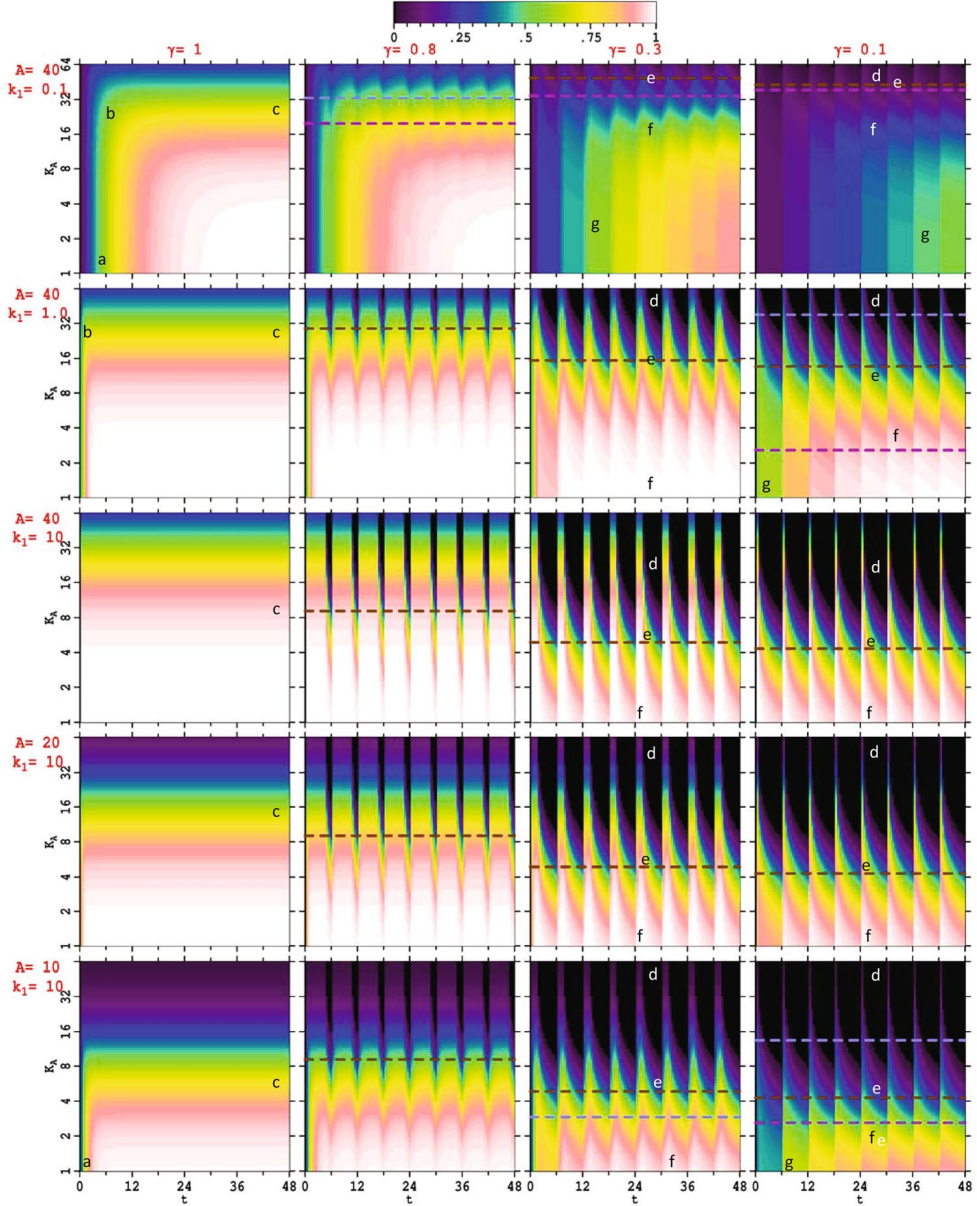
Promoter binding probabilities *P*(*t*) (*t* in h) for *n* = 2 shown over the range of 1–64 nM in dissociation constants *K*_*A*_, for various values of *A* (in nM) and *k*_1_ (in 10^−3^ nM^−2^ h^−1^), rows, and for various duty cycles γ (γ = 1 indicates sustained signaling), columns. The pulsing period is *T* = 6 h. The dashed lines indicate, when feasible, the maximum values of *K*_*A*_ satisfying inequalities Eqn. (9) (brown, fast pulsing limit), Eqn. (22) (grey, pulsing slows rise time), and Eqn. (24) (magenta, clocking such that binding increases with each pulse). Marked regimes are: *a*-*K*-independent initial binding, *b*-changeover, *c*-asymptotic regime, *d*-slow pulsing, *e*-competitive pulsing, *f*-fast pulsing, *g*-graded activation per pulse.

Numerically obtained solutions are displayed in 20 two-dimensional images in Fig. 3 for *n* = 2, color coded with black corresponding to no binding and white to 100% binding probability. Provided that the Hill coefficient *n* is near 2 or greater, qualitative scenarios do not depend greatly on its value; we present results for *n* = 4 in SI Supplementary Figure S1 but otherwise do not explore this aspect. In each image of Fig. 3, the ordinate specifies *K*_*A*_ selected logarithmically in the range of 1–64 nM with all other parameters held constant. This range is of the order of that anticipated in many biochemical signaling situations and is chosen purely for convenience. In all scenarios presented, a clocking period of *T* = 6 h is used, also typical of many biochemical rhythms (e.g., circadian rhythms of 24 h and observed p53 clocking periods of the order 5–8 h^1,4,41,42,54,70–73^). Adaption of the results presented to situations with variant signal-molecule maximum concentrations and variant periods is straightforward as these parameters basically act to scale the time and concentration scales of interest rather than influence the underlying chemical scenarios. Given these choices, to expose basic Hill-type pulsed signaling scenarios, the 20 images shown are produced considering indicative values of the other parameters *A* = 10, 20 or 40 nM and *k*_1_ = 0.1, 1 and 10 × 10^−3^ nM^−2^ h^−1^, as well as the cases γ = 1 (sustained signaling), 0.8 (weak pulsing), 0.3 (typical of p53 pulsed signaling scenarios^1,4,41,42,54,70–73^), or 0.1 (strong pulsing). For reference, the corresponding values of *k*_2_ at each point on these figures from Eqn. (3) are shown in Supplementary Figure S2a and range enormously from 10^−4^ h^−1^ to 10^3^ h^−1^. Also, some characteristic individual traces of *P*(*t*) at *K*_*A*_ = 2, 8, 32 and 64 nM extracted from this figure are shown in Supplementary Figure S3a, mapping the color coding used in Fig. 3 onto a more conventional one-dimensional representation of *P*(*t*).

In Fig. 3, the first column of figures depicts promoter binding scenarios associated with sustained signaling. As indicated by Eqn. (4), the initial binding rate is controlled by *k*_1_ and *A* and is independent of *K*_*A*_. Such regimes of independence of the dynamics on the dissociation constant are marked *a* on the figure. At long times, the binding approaches an asymptotic value ${\bar{P}}_{sus}$ which does depend on *K*_*A*_ (Eqn. (8)). These asymptotic regimes are marked *c* while the changeover region between the short-time and long-time dynamics is marked *b*.

The subsequent columns in Fig. 3 indicate progressively increasing pulsing. Regimes analogous to *a*, *b* and *c* remain but only new features associated with the pulsing are marked. Pulsed signaling always reduces the binding compared to sustained signaling at the same peak signal-molecule concentration *A*, but the magnitude of the reduction is strongly dependent on the binding affinity and rate constants and oscillates in synchronization with the pulsing. Pulsing effects take on three fundamental forms depending on the frequency of the pulsing compared to the speed at which the chemical reactions take place and are categorized into the regimes:

9 $$\begin{array}{cc}(1-\gamma )T\gg 1/{k}_{2}: & d-\quad {\rm{s}}{\rm{l}}{\rm{o}}{\rm{w}}\,{\rm{p}}{\rm{u}}{\rm{l}}{\rm{s}}{\rm{i}}{\rm{n}}{\rm{g}},\\ (1-\gamma )T\approx 1/{k}_{2}: & e-\quad {\rm{c}}{\rm{o}}{\rm{m}}{\rm{p}}{\rm{e}}{\rm{t}}{\rm{i}}{\rm{t}}{\rm{i}}{\rm{v}}{\rm{e}}\,{\rm{p}}{\rm{u}}{\rm{l}}{\rm{s}}{\rm{i}}{\rm{n}}{\rm{g}},\\ (1-\gamma )T\ll 1/{k}_{2}: & f-\quad {\rm{f}}{\rm{a}}{\rm{s}}{\rm{t}}\,{\rm{p}}{\rm{u}}{\rm{l}}{\rm{s}}{\rm{i}}{\rm{n}}{\rm{g}}.\end{array}$$

In region *f* the pulses happen faster than the biochemical reactions can respond so that variations of the binding within each pulse period are small. However, the amount of binding found asymptotically at long times can vary considerably, from nearly that expected for sustained signaling to almost nothing at all. Alternatively, when the chemical reactions occur quickly on the time scale of each pulse, the signal-on part of the pulse sees the binding rise to near its level for sustained pulses and then fall to near zero during the signal-off part of the cycle, making the bound concentration parallel the signal-molecule concentration at each instant in time. This is the slow pulsing limit, regime *d*. Between these two regimes occurs the situation in which the changes in binding caused by the biochemical reaction rates compete with those caused by the pulsing of the signal molecule, regime *e*, the loci of which are marked by brown dashed lines on Fig. 3.

As ${k}_{2}={K}_{A}^{n}{k}_{1}$ (Eqn. (3)), with all other parameters fixed, reactions of sufficiently low affinity (high *K*_*A*_) will always be able to access the slow pulsing regime in which the binding simply follows the pulsing. Also, reactions of sufficiently high affinity (low *K*_*A*_) will always be able to minimize the effects of pulsing to drive complete binding, especially at longer times. Apparent from Fig. 3 though, the regime *e* of competitive dynamics often but not always demarks the changeover between the low-affinity and high-affinity scenarios, but in any case it is clear that these limits do merge smoothly together.

From a qualitative perspective, other important aspects of pulsed dynamics revealed in Fig. 3 are ways in which the binding is controlled in some way by clocking. In regime *d*, the binding simply follows the pulsing and so the clocking aspects of the signal molecule concentration are just purveyed to the bound product^22,23,48,52^. However, within domains of regime *f* marked *g*, another type of clocking is found: the level of binding is held fixed during each pulse, but this level increases linearly with the number of pulses. Both types of clocking allow for fine control of biochemical systems.

### Sensitivity of low-affinity reactions to signal-molecule concentration

An important feature of Hill-type dynamics revealed is that for low-affinity promoters with ${K}_{A}^{n}\gg {A}^{n}$, the asymptotic binding becomes very sensitive to signal-molecule concentration, scaling for sustained signaling from Eqn. (8) as

10 $${\bar{P}}_{sus}\approx \frac{{A}^{n}}{{K}_{A}^{n}}.$$

This effect of sustained signaling underlies pulsed signaling and gives rise to the large changes in qualitative appearance between the results shown in Fig. 3 at *k*_1_ = 10 × 10^−3^ nM^−2^ h^−1^ for *A* = 10, 20, and 40 nM.

### Suppression of low-affinity reactions

If the affinity of the signal molecules for the receptor is so low that the slow pulsing limit (Eqn. (9)) applies, then the binding can be assumed to respond instantly to changes in signal-molecule concentration making

11 $$P(t)=\frac{[{\rm{S}}]}{A}.$$

Hence the ratio of the average level of binding with signal pulsing after infinite time to that without (Eqn. (8)) is given by

12 $$\frac{{\bar{P}}_{pulsed}}{{\bar{P}}_{sus}}=\gamma .$$

In SI, equations for the opposite fast-pulsing limit are derived, yielding

13 $$\frac{{\bar{P}}_{pulsed}}{{\bar{P}}_{sus}}=\gamma \frac{{K}_{A}^{n}+{A}^{n}}{{K}_{A}^{n}+\gamma {A}^{n}},$$

which approaches unity for high affinity ligands while approaching the same result, γ, for low affinity ligands. Hence independent of the pulsing period, low-affinity reactions are suppressed by pulsed signaling, with the extent of the depression scaling with the duty cycle; the shorter the duty cycle, the greater the effect. In absolute terms, the asymptotic average binding for pulsed signaling in the fast-pulsing limit can be written as

14 $${\bar{P}}_{pulsed}=\frac{\gamma {A}^{n}}{{K}_{A}^{n}+\gamma {A}^{n}}=\frac{{\overline{[{\rm{S}}]}}^{n}}{{K^{\prime} }_{A}^{n}+{\overline{[{\rm{S}}]}}^{n}},$$

where from Eqn. (1)

15 $$\overline{[{\rm{S}}]}=\gamma A$$

is the average signal-molecule concentration and ${K^{\prime} }_{A}$ is the apparent dissociation constant, i.e., the average concentration of the signal molecule at which half of their receptors are fully complexed. This is reduced from the concentration required to complex half of the receptors for sustained signaling *K*_*A*_ by the factor

16 $$\frac{{K^{\prime} }_{A}}{{K}_{A}}={\gamma }^{(n-1)/n}.$$

**Figure 4:**
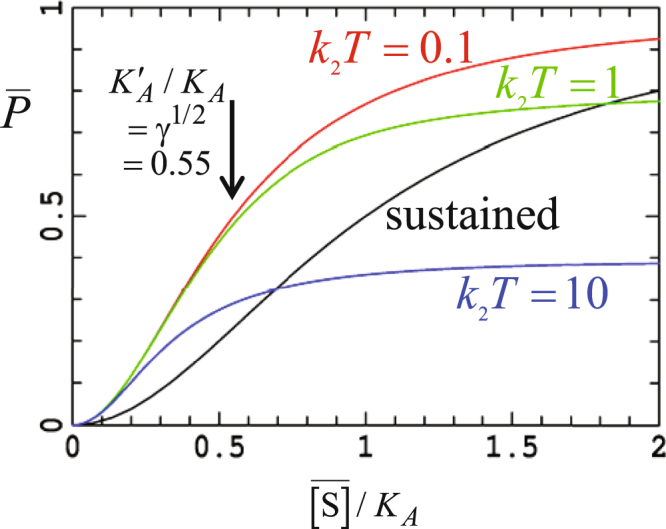
Average binding probability at infinite time as a function of the average signal-molecule concentration when *n* = 2 for sustained signaling and for pulsed signaling at γ = 0.3 with *k*_2_*T* = 0.1 (fast pulsing), 1, and 10 (slow pulsing).

This effect has been observed for calcium signaling^12^ and is understood^14,15^. When *n* = 2 the apparent dissociation constant reduced by a factor of γ^1/2^, enhancing to γ^3/4^ for *n* = 4. This effect is illustrated in Fig. 4 where “titration curves” giving the average binding as a function of average signal-molecule concentration are shown for γ = 0.3 and *n* = 2; in the fast-pulsing limit *K*_2_*T*=0.1, the reduction factor for the apparent dissociation constant is (0.3)^1/2^ = 0.55, becoming (0.3)^3/4^ = 0.41 if *n* is equal to 4.

Results are included in Fig. 4 also for competitive pulsing (*K*_2_*T*=1) and for slow pulsing (*K*_2_*T*=10). The reduction in the apparent dissociation constant is seen to be universally maintained, with Eqns (12) and (13) merging smoothly together. The origin of this effect is in the requirement that multiple signal molecules must bind to the receptor, and as a consequence short time exposure at high signal-molecule concentration is more effective than long-time exposure at an averaged concentration. Numerical results for a sinusoidal pulsed Hill model have also focused on this feature and its chemical origin^8^, a feature observed also in extensive numerical simulations of p53 dynamics^8^. As a result, *pulsed signaling enhances the roles of receptors having high binding affinity by reducing the average signal-molecule concentration required for activation*. Another way of considering this is through the ratio of the binding from sustained signaling to that from pulsed signaling at the same averaged signal-molecule concentration,

17 $$\frac{{\bar{P}}_{sus}({\rm{a}}{\rm{t}}\,[{\rm{S}}]=\gamma A)}{{\bar{P}}_{pulsed}}={\gamma }^{n-1}\frac{{K}_{A}^{n}+\gamma {A}^{n}}{{K}_{A}^{n}+{\gamma }^{n}{A}^{n}}$$

which approaches unity as γ→1 but decreases rapidly to order γ^*n*−1^ as γ→0.

Of interest also is the accumulated product ${c}^{{\rm{P}}}(t^{\prime} )={\int }_{0}^{t^{\prime} }P(t){\rm{d}}t$ and accumulated signal molecule ${c}^{{\rm{S}}}(t^{\prime} )={\int }_{0}^{t^{\prime} }[{\rm{S}}]{\rm{d}}{\rm{t}}$ levels over time. We consider the ratio of cumulative product obtained from sustained and pulsed signaling at the *different* times needed to make the *same* accumulated signal-molecule exposure ${c}^{{\rm{S}}}\equiv {c}_{sus}^{{\rm{S}}}(t^{\prime} )={c}_{pulsed}^{{\rm{S}}}(t^{\prime\prime} )$, as discussed in the SI. Asymptotically, this ratio becomes

18 $$\mathop{{\rm{l}}{\rm{i}}{\rm{m}}}\limits_{{c}^{s}\to {\rm{\infty }}}\frac{{c}_{sus}^{P}({c}^{s})}{{c}_{pulsed}^{P}({c}^{s})}=\{\begin{array}{cc}1 & {\rm{f}}{\rm{o}}{\rm{r}}\,{\rm{s}}{\rm{l}}{\rm{o}}{\rm{w}}\,{\rm{p}}{\rm{u}}{\rm{l}}{\rm{s}}{\rm{i}}{\rm{n}}{\rm{g}}\\ \frac{{K}_{A}^{n}+\gamma {A}^{n}}{{K}_{A}^{n}+{A}^{n}} & {\rm{f}}{\rm{o}}{\rm{r}}\,{\rm{f}}{\rm{a}}{\rm{s}}{\rm{t}}\,{\rm{p}}{\rm{u}}{\rm{l}}{\rm{s}}{\rm{i}}{\rm{n}}{\rm{g}}\end{array}$$

which approaches 1 for low-affinity reactions and γ for high-affinity reactions. Therefore, for either slow pulsing or low-affinity reactions, only the total amount of signal-molecule delivered is important and signal pulsing has no unique biochemical outcome. Alternatively, for fast-pulsed high-affinity reactions, a small amount of signal-molecule exposure creates the same amount of accumulated product as is produced by a large amount of the signal molecule applied with sustained signaling.

### Fine control of low-affinity ligand activation by clocking

Both Eqns (12) and (13) show the average promoter binding of low affinity ligands scaling with γ, independent of the clocking period. As the duty cycle is a property typically able to be controlled in biochemical applications, this allows reactions to be controlled. Alternatively, Eqn. (8) shows how such reactions can be controlled through modulation of the signal-molecule peak concentration *A*. The significant practical difficulty in utilizing concentration to control activity in this way is indicated in Fig. 3 where results for *A* = 40, 20 and 10 nM at *k*_1_ = 10 × 10^−3^ μM^−2^ h^−1^ are shown; for a low-affinity ligand with *K*_*A*_ = 32 nM, the sustained-signaling binding probabilities change from 61% to 28% to 0.9%, respectively, owing to the sensitivity of Eqns (8) and (10) to concentration. At *n* = 4, the changeover would be faster, with the analogous probabilities becoming 71%, 13%, and 0.9% (see SI Supplementary Figure S1). In any case, this scenario demands tight control of sustained-signaling concentrations in order to regulate the activity of low-affinity ligands. Through Eqns (12) and (13), this sensitivity is transferred also to pulsed signaling, but the key feature here is that pulsed signaling can *ensure* suppression of low-affinity reactions when sustained signaling cannot.

### Slowing down of high-affinity reactions

By scanning the top two rows in Fig. 3 (see also Supplementary Figure S3a), it is clear that increasing pulsing can delay the binding of high-affinity promoters. The rise times for attaining the steady-state averaged promoter bindings $\bar{P}$ (see SI) are

19 $${\tau }_{sus}=\frac{1}{{k}_{1}({A}^{n}+{K}_{A}^{n})}=\frac{1}{{k}_{1}{A}^{n}+{k}_{2}}$$

for sustained signaling, and as is known^15^ in regime *f* for fast pulsing (Eqn. (9)),

20 $${\tau }_{pulsed}=\frac{1}{{k}_{1}({A}^{n}\gamma +{K}_{A}^{n})}-\frac{T}{2}(1-\gamma ).$$

Both lifetimes grow large as both *A*^*n*^ and ${K}_{A}^{n}$ become small, but the most interesting regime is the one in which the pulsed lifetime is the longest. This occurs whenever

21 $$T < \frac{2{k}_{1}{A}^{n}}{({k}_{1}{A}^{n}+{k}_{2})({k}_{1}{A}^{n}\gamma +{k}_{2})}=\frac{2{A}^{n}}{{k}_{1}({A}^{n}+{K}_{A}^{n})({A}^{n}\gamma +{K}_{A}^{n})}$$

or equivalently, when

22 $${K}_{A} < \frac{A}{{(2\gamma )}^{1/n}}{\{{[{(1-\gamma )}^{2}+\frac{8}{{k}_{1}T{A}^{n}}]}^{1/2}-(1+\gamma )\}}^{1/n}.$$

The maximum values of *K*_*A*_ for which this holds are indicated on Fig. 3 by gray dashed lines; when no line appears on a figure, no (real) values of *K*_*A*_ are able to satisfy the equation and so binding is *never* significantly delayed. When it is satisfied, sometimes the value of *K*_*A*_ exceeds that needed to achieve fast pulsing (brown dashed lines on figure), an assumption used in the derivation of Eqn. (22), and therefore in this case fast pulsing *always* significantly delays binding. When binding is delayed, for small enough pulsing periods *T*, the increase in the rise time is by a factor of

23 $$\frac{{\tau }_{pulsed}}{{\tau }_{sus}}=\frac{{A}^{n}+{K}_{A}^{n}}{{A}^{n}\gamma +{K}_{A}^{n}}\to 1+\frac{{A}^{n}}{{K}_{A}^{n}}\,{\rm{as}}\,\gamma \to {\rm{0}}{\rm{.}}$$

This ratio grows large whenever ${A}^{n}\gg {K}_{A}^{n}$, i.e., when the binding affinity and/or the peak concentration are high.

### Graded activation of high-affinity ligands by clocking

In the fast-pulsing regime where binding is significantly delayed (small oscillations satisfying Eqn. (22)), regimes *g* in Fig. 3 show a clocking scenario in which the level of bound promoter jumps discontinuously on each successive pulse. As shown in SI, the first cycle in general produces a relatively small amount of binding ${P}_{1}(T)\ll {\bar{P}}_{pulsed}$ whenever

24 $${K}_{A}^{n}\ll \frac{1}{{k}_{1}T}-\gamma {A}^{n}\,{\rm{or}}\,{k}_{2}T\ll 1-{k}_{1}{A}^{n}\gamma T,$$

a condition indicated by purple dashed lines on Fig. 3 that is stronger than that required for fast pulsing ((1−γ)*k*_2_ ≪ 1 from Eqn. (9), shown on figure as brown dashed lines). Then, in the short-time linear regime of the pulse-averaged dynamics, the binding produced after the *i-*th pulse *P*_*i*_(*T*) simply increases initially in proportion to the number of pulses received:

25 $${P}_{i}(T)\approx i{P}_{1}(T).$$

In regime *g*, each pulse thus triggers the same *increase* in promoter activity, with receptor binding increasing in proportion to the number of signal pulses. From Eqn. (24), this regime only exists whenever

26 $${k}_{1}{A}^{n}\gamma T\ll 1.$$

### Developing an application to understanding p53 signal decoding

For S = p53 binding to R = all DNA genes of current relevance, the number of molecules that must bind is four^45,61–63^. However, the way that this binding occurs is critical to kinetic analyses and hence the perceived Hill coefficient^32–34^. Many different scenarios for this could be conceived, as discussed in SI, leading to effective Hill coefficients in the range *n* = 1 to 4. In different applications of pulsed Hill-type signaling, these scenarios could apply. In brief, applied to p53, one scenario is cooperative binding in which the first p53 molecule binds weakly but activates binding for the remaining molecules, leading to a Hill coefficient of *n* = 4. Another conceivable mechanism is full pre-association of p53 into a tetramer and then the binding of the tetramer onto the DNA. If the tetramer concentration is small, as envisaged, then the appropriate Hill coefficient would again be *n* = 4, otherwise if p53 fully tetramerized under all reaction conditions of interest then it would be *n* = 1. However, while involvement of pre-assembled tetramers^74–77^ is recognized, measurements of binding of p53 to wild-type DNA *in vitro*^62^ indicates a Hill coefficient of *n* = 1.8~2. This is usually interpreted as involving the dimerization reaction

27 $$2{\rm{p53}}\underset{{k}_{2d}}{\overset{{k}_{1d}}{\rightleftarrows }}{{\rm{p53}}}_{2}\quad \quad {K}_{Ad}=\frac{{k}_{2d}}{{k}_{1d}}=\frac{{[{\rm{p53}}]}^{2}}{[{{\rm{p53}}}_{2}]}$$

followed by cooperative receptor binding

28 $$2{{\rm{p53}}}_{2}+{\rm{DNA}}\underset{{k}_{2R}}{\overset{{k}_{1R}}{\rightleftarrows }}{\rm{DNA}}\mathrm{.4p53},\quad \quad {K}_{AR}^{2}=\frac{{k}_{2R}}{{k}_{1R}}.$$

It is possible to obtain analytic solutions to these coupled equations. By analogy to Eqn. (8), the asymptotic binding probability for sustained signaling becomes

29 $${\bar{P}}_{sus}=\frac{{[{{\rm{p53}}}_{2}]}^{2}}{{K}_{AR}^{2}+{[{{\rm{p53}}}_{2}]}^{2}}.$$

Ignoring the amount of p53 bound to the DNA, the mass balance equation accounting for the total p53 concentration [p53]_total_ becomes

30 $${[{\rm{p53}}]}_{{\rm{total}}}=[{\rm{p53}}]+2[{{\rm{p53}}}_{2}]$$

so that from Eqn. (27),

31 $$8[{{\rm{p53}}}_{2}]=4{[{\rm{p53}}]}_{{\rm{total}}}+{K}_{Ad}-{({K}_{Ad}^{2}+8{K}_{Ad}{[{\rm{p53}}]}_{{\rm{total}}})}^{1/2}.$$

In the limits of strong and weak dimerization, Eqn. (29) therefore yields

32 $$\begin{array}{c}{K}_{Ad}\ll 4{[{\rm{p53}}]}_{{\rm{total}}}:\quad \quad {\bar{P}}_{sus}=\frac{{[{\rm{p53}}]}_{{\rm{total}}}^{2}/4}{{K}_{AR}^{2}+{[{\rm{p53}}]}_{{\rm{total}}}^{2}/4},\,{\rm{and}}\\ {K}_{Ad}\gg 4{[{\rm{p53}}]}_{{\rm{total}}}:\quad \quad {\bar{P}}_{sus}=\frac{{[{\rm{p53}}]}_{{\rm{total}}}^{4}/{K}_{Ad}^{2}}{{K}_{AR}^{2}+{[{\rm{p53}}]}_{{\rm{total}}}^{4}/{K}_{Ad}^{2}},\end{array}$$

respectively. Hence for strong dimerization the kinetics takes the form of a Hill equation with *n* = 2, while for weak dimerization it is of the form with *n* = 4. The observed^62^ coefficient of *n* = 1.8 is interpreted as indicating that p53 is dimerized under most conditions of interest. Measured values of *K*_*AR*_ for wild-type p53 *in-vitro* are listed in SI Supplementary Table S1 and subsequently interpreted in terms of the Hill mechanism, Eqn. (2), using

33 $$R=\mathrm{DNA},\,{\rm{S}}={{\rm{p53}}}_{2},\,n=2,\,{K}_{A}={K}_{AR},\,{\rm{and}}\,A={[{\rm{p53}}]}_{{\rm{total}}}/2.$$

While this application was obtained by forcing a simple Hill equation to mimic the the equilibrium state of a complicated system, the way in which equilibrium is reached may be depicted quite differently. If the rate constants *k*_1*d*_ and *k*_2*d*_ depict faster dimerization equilibration than *k*_1*R*_ and *k*_2*R*_ depict receptor binding, then indeed Eqn. (4) will provide a realistic approximation to the kinetics of the complex system. However, if the dimerization reaction is relatively slow then these properties would significantly affect pulsed signaling responses. Accurate calculations therefore need to include all implicated chemical processes directly, but determining accurately the required parameters may not be easy. In discussions of p53 binding, fast rates for constants *k*_1*d*_ and *k*_2*d*_ are usually assumed, and we precede making this assumption to allow simple Hill-type kinetics schemes to be applied.

Normal cells not subject to large external radiation or chemotoxic stresses have low levels of p53^41,42,54,78^. After cell damage caused by gamma radiation, temporal variations in p53 concentration cause the expression of e.g., the p21, GADD45A and XPC genes, initiating cell cycle arrest and DNA repair^79^, as has been recently reviewed^10^. During this process the active-p53 level is pulsed; the pulses have constant amplitude and duration but the probability and number of pulses increases with the radiation dose received^42,80^. Though individual cells show significant variations^71^, the effect is widely observed^41,73,81,82^. However, in addition to the expression of these genes, p53 also activates expression of BAX, PUMA, Noxa and other genes that, after a certain length of time, results in apoptosis or senescence^66,83^, perhaps aided by conversion of the pulsed p53 signal into a sustained one^10,52^. Alternatively, cell exposure to ultraviolet radiation results in the generation of a single sustained p53 pulse^84^, expressing apoptosis genes as well as binding to Bcl-2 to interfere with anti-apoptosis mechanisms in the mitochondria to induce rapid cell death^9,84–87^. Also, exposure to high doses of chemotherapy agents results in concentrated, continually rising p53 levels^88,89^. A significant feature relating to p53 function is the observation that the function of the proteins expressed by the PUMA, TP53INP1 and other related apoptosis genes is subject to a threshold mechanism^29,66,83^, as well as the observation that the intrinsic lifetime of produced mRNA is of importance^43^.

However, we focus on a key observation concerning p53 decoding obtained using targeted perturbation technology that artificially changes p53 concentrations dynamically^4^. In this process, a *pulsed* active-p53 signal triggered by gamma irradiation was artificially changed into a *sustained* signal at various levels of total p53 exposure. Results indicated that, independent of the total exposure, pulsed signaling led to expression of cell-cycle arrest (CDKN1A) and repair (GAD45A) genenes whereas sustained signaling led to activation of genes related to apoptosis (BAX) and senescence (PML, YPEL3). The cellular impact of the expressed senescence genes was found to be more profound than that for BAX, perhaps owing to the complexity of its interactions^10^. As no other biochemical processes were altered in this procedure except p53 signal pulsing and the effect is independent of total p53 dosing, it is clear that the *pulsing* of the signal is critical to the decoding mechanism, not just the peak or average p53 concentrations or external effects such as post-translational modifications. Although suggestions have been made^4^, accounting for this non-linear dynamical effect by the current very complex models of p53 dynamics^9,10^ has not been properly addressed, though mRNA lifetime is known to be a contributing factor^43^.

Development of a simple pulsed-signaling Hill-type model to interpret this data requires many assumptions. While different DNA binding scenarios lead to different Hill coefficients, we adopt the strong dimerization model of Eqn. (33) involving *n* = 2. This is not a critical assumption, however, as the basic scenarios associated with pulsed signaling are insensitive to *n* in the range of 2–4. We proceed assuming that the wild-type *in-vitro* binding affinities measured for p53 binding to various gene promoters^68,69^ (see Supplementary Information Table S1) provides a robust description. Unfortunately little is known of a key feature required for model application, i.e. concentration of the active oligomerized form of p53 *in vivo* in stressed cells; related data is only available for total p53 levels in *unstressed* cells and spans a very wide range of^45^ 60–500 nM.

### A possible role for pulsed Hill-type dynamics for p53?

The results presented earlier in Fig. 3 pertain to p53 signal decoding for *n* = 2, with very similar results presented in SI Supplementary Figure S1 for *n* = 4. How DNA binding by p53 relates to the identified scenarios is unknown, but we proceed seeking feasible options that can qualitatively interpret key data.

Data^4,43^ comparing the time dependence of system responses to relative p53 levels clearly indicates that p53 binds to most gene promoters in either regime *d* or *e*. In the slow-pulsing regime *d*, the time dependence of the signal molecule is simply transferred to give the time dependence of bound DNA promoters. However, in the competitive pulsing regime *e*, promoters remain bound for some time after the p53 signal is reduced, enhancing gene activity. Of particular interest is the cell-cycle arrest gene CDKN1A (p21) which has a much higher binding affinity (*K*_*Ad*_ = 5–12 nM) than most other genes. Could the other genes be in regime *d* whilst it is in regime *e*? In regime *e*, pulsing enhances outcomes compared to sustained signaling, a key effect observed for CDKN1A by Purvis *et al*.^4^, but in regime *d* pulsing has minimal effect.

Another feature observed by Purvis *et al*.^4^ is that the same amount of cumulative p53 obtained using sustained signaling induces more protein synthesis than does that amount coming from pulsed signaling, especially for the high-affinity CDKN1A cell-cycle arrest gene^4^. However, this result is opposite to that indicated by Eqn. (18), which instead predicts that the sustained to pulsed ratio approaches γ for fast-pulsed high-affinity ligands and should always be *less* than one. This difference between prediction and expectation could arise from fine details in the experimental procedure. While experimental conditions were designed to produce signaling with the p53 concentration sustained at the maximum amplitude of the pulsed signaling, the actual p53 concentrations obtained for p21 were about twice as large (ref.^4^ Fig. 2A). Given this variation, Eqn. (18) is not valid as a second effect, enhancement of the sustained binding owing to signaling amplitude fluctuations (Eqn. (8)), is also operative. In the limit of slow pulsing (regime *d*), doubling the sustained p53 concentration would enhance binding by a factor of 2^*n*^ (Eqn. (10)) at only a cost of a factor of 2 in cumulative p53, making Eqn. (18) fall short by a factor of 2^*n*−1^ = 2. This effect continues also into the competitive pulsing domain, regime *e*, but only when *A* is of the order of the dissociation constant for p21, 5–12 nM.

While concentrations of the active form of p53 of the order of 5–12 nM are dramatically less than the observed total p53 levels in unstressed cells^45^, the recent extensive model simulations of Liu *et al*.^9^ deduced a value of *A* = 6 nM. Figure 3 shows results for *A* = 10, 20, and 40 nM for *k*_1_ = 10 × 10^−3^ M^−2^ h^−1^; we focus on results for γ = 0.3. If the cell-cycle arrest gene CDKN1A lies in regime *e*, while lower-affinity genes associated with apoptosis like BAX and TP53AIP1 lie in regime *d*, then the important result^4^ that pulsing p53 leads to preferential enhancement of p53 is immediately understood.

A significant aspect of the pulsed Hill-type signaling model is the prediction of a novel clocking mechanism of regime *g* in which incrementally more binding of low-affinity promoters occurs with each pulse. Accessing this regime would require higher binding affinity than any of the discussed gene promoters and hence does not at present appear to play any role in decoding p53 dynamics. However, Fig. 3 shows that regime *g* could be accessed if the ligand binding affinity and pulsing could be increased (e.g., for *K*_*A*_ = 1 nM, *k*_1_ = 10 × 10^−3^ M^−2^ h^−1^, γ = 0.1). Experimental studies^4,43^ would be able to detect this regime by noting that protein yields increased non-linearly with time, even within regimes in which the p53 levels were low. Nevertheless, many other biological signaling processes, for example calcium signaling, could easily display this effect.

## Discussion

Hill-type decoding functions for pulsed signaling are shown to lead to four significant modifications to conventional signaling. These are: the suppression of low-affinity reactions, the ability to control these suppressed reactions using clocking to limit activation, the slowing down of the rate at which high-affinity reactions occur, and the possibility of a unique clocking domain in which high-affinity reactions are driven further with each successive pulse.

Concerning p53 decoding, most gene promoters appear to bind p53 in the slow-pulsing regime *d* in which no novel effects are associated with signal pulsing. However concerning CDKN1A which binds with much higher relative affinity, regime *e* could be accessed and explain the observed relative enhancement of this gene induced by signal pulsing. If this were so then the very unusual regime *g* of Hill-type dynamics could be accessed by further increasing the binding affinity; within regime *g* each successive pulse simply increases the binding incrementally. However, before this effect occurs, the fast-pulsing regime *f* would be reached in which the binding no longer decays during the part of the pulsing cycle in which the p53 level reduces. Discovery of these predicted effects would significantly aid understanding of p53 decoding.

From the diversity of scenarios that can result from pulsed Hill-type signaling and the large differences of many of these from sustained signaling, to be authoritative all modeling schemes must fully include the involved chemical processes. While in the field of p53 research only a few models ignore pulsed Hill-type signal decoding features^9,49–51,55,57^, in those that do include them^8,48,52–54,56,57^ it is not clear whether the correct parameters are used or the deduced properties can be simply attributed to the Hill-type features. Different models adopt very different values for key quantities, with, e.g., the Hill coefficient *n* itself often specified as 2, 3, or 4 in classic works^58–60^ and those that follow them. Authoritative studies demand knowledge of the active-p53 concentration, but in many the actual scale for p53 concentration is not even specified^4,8,37,49,50,52–55,90^. Alternatively, in simulations embodying *measurable* parameters, the concentrations used for the *active* form of p53 in *stressed* cells include 1000–5000 nM^89^, 750 nM^51^, 300 nM^91^ and 6 nM^9^. This range extends from much-less-than to much-greater-than the measured p53 concentration in unstressed cells (60–500 nM^45^), and when combined with Hill coefficients of order *n* = 2–4 depicts a range of promoter binding rates varying by a factor of 10^6^–10^12^! To provide a rigorous platform for future modeling, basic issues like these need to be resolved, leading to better understanding of all observed effects including the effect of pulsing on signal decoding.

While discussion has focused on p53 decoding, Hill-type scenarios are widely applicable to biochemical signal decoding, and it is likely that the properties described can be identified in many other systems. This includes systems well studied by Hill-type mechanisms such as calcium signaling^14,15^ and circadian rhythms^17^ but is likely to involve also many more.

## Methods

The methods used involve analytical mathematical equation solution, followed by numerical simulations of the resulting equations performed using FORTRAN.

### Data availability

All data is described in the text and SI. The simulation software is available on request from the authors.

## Electronic supplementary material

Supplementary Information
