# Supplementary material for: Understanding non-linear effects from Hill-type dynamics with application to decoding of p53 signaling
Source: Sci Rep. 2018 Feb 1;8:2147. doi: 10.1038/s41598-018-20466-2 (PMC5795017; doi:10.1038/s41598-018-20466-2)
Supplement: Supplementary file 1 — Supplementary Information [file 41598_2018_20466_MOESM1_ESM.pdf]

Understanding non-linear effects from Hill-type dynamics with application to decoding of p53 signaling

Xiaomin Shi[1]* and Jeffrey R. Reimers[2,3]*

[1] *International Centre for Quantum and Molecular Structures and Mathematics Department, Shanghai University, Shanghai 200444 China*

[2] *International Centre for Quantum and Molecular Structures and Physics Department, Shanghai University, Shanghai 200444 China*

[3] *School of Mathematical and Physical Sciences, University of Technology Sydney, NSW 2006, Australia*

* shixm@shu.edu.cn, reimers@shu.edu.cn, jeffrey.reimers@uts.edu.au

Supplementary Results

**S1**	Model variable and parameter definitions	2
**S2**	Binding affinities of p53 to gene promoters	3
**S3**	Probability of the signal molecule binding to the receptor	3
**S4**	The relationships between the steady mean binding probability, the average signal-molecule concentration, and the duty cycle	10
**S5**	Times to reach steady-state binding driven by pulsed and sustained signaling	11
**S6**	The relationship between binding probability and number of pulses during the initial stages of relaxation	12
**S7**	Cumulative signal molecule exposure and cumulative binding	13
**S8**	Some other ways in which Hill-type signaling can manifest when 4 signal molecules must bind to the receptor a la p53 binding	16
**S9**	Supplementary References	18

S1. Pulsed Hill-model: variable and parameter definitions

Symbol	Meaning	Units
$[\mathrm{S}]$	time-dependent signal-molecule concentration, controlled by encoder	nM
$\overline{[\mathrm{S}]}$	averaged signal-molecule concentration at long time, pulsed signaling	nM
A	maximum signal mol. concentration, pulsed (or sustained) signaling	nM
$[\mathrm{RS}_n]$	concentration of fully formed complex of signal molecule and receptor	nM
$[\mathrm{R}]$	concentration of receptor molecule	nM
n	Hill coefficient, number of signal molecules that must bind in order to activate the receptor	-
$P(t)$	general probability that receptors are fully complexed	-
$P_{sus}(t)$	probability that receptors are fully complexed under sustained signaling	-
$P_i(\xi)$	probability that the receptor is fully complexed under pulsed signaling during the *i*-th cycle at relative time	-
$\bar{P}_i$	average binding probability during *i*-th cycle, pulsed signaling	-
$\bar{P}_{pulsed}$	asymptotic steady-state averaged binding probability per period, pulsed signaling	-
$\bar{P}_{sus}$	asymptotic steady state binding probability, sustained signaling	-
k_1	rate constant for association	$\mathrm{nM}^{-n}\mathrm{h}^{-1}$
k_2	rate constant for disassociation	h^{-1}
K_d	disassociation equilibrium constant = k_2/k_1	nM^n
K_A	dissociation constant ([S] at which equal amounts of complexed and uncomplexed receptors are present for sustained signaling) = $K_d^{\ 1/n}$	nM
K'_A	apparent dissociation constant ([S] at which equal amounts of complexed and uncomplexed DNA are present for pulsed signaling)	nM
t	time	h
ξ	time relative to the start of a cycle	h
T	period of pulsed signaling	h
Δ	time during each period that the signal molecule is present	h
γ	duty cycle = Δ / T	-
τ_{sus}	relaxation time to steady state for sustained signaling	h
τ_{pulsed}	relaxation time to steady state for pulsed signaling	h

S2. Binding affinities of p53 to gene promoters

Supporting Table 1. Averaged values for the in-vitro dissociation constants K_{Ad} of wild-type p53 dimers to DNA promoters, from Weinberg et. al.[1].

gene promoter	K_{Ad} / nM	affinity description
CDKN1A (p21) site 5′	4.9 ± 0.6	high
CDKN1A (p21) site 3′	12.0 ± 7.0	medium
GADD45A (GADD45)	7.7 ± 1.2	medium
PUMA BS2	7.1 ± 1.8	medium
PUMA BS1	260 ± 50	very low
Noxa	8.6 ± 1.5	medium
TP53AIP1	11.0 ± 1.8	medium
BAX	73 ± 33	low
MDM2	12.9 ± 2.9	medium
PCNA	6.6 ± 1.4	high
IGF-BP3 Box A	81 ± 25	low
TP53INP1 (P53DINP1)	98 ± 23	low

S3. Probability of the signal molecule binding to the receptor.

This analysis is an adaptation of that developed for calcium signaling[2]. Equation (5) from the main text indicates that the binding probability $P(t)$ satisfies

$$\frac{dP(t)}{dt} = \left(1 - P(t)\right) k_1 \left[\mathrm{S}\right]^n - k_2 P(t) \ , \tag{5}$$

where $\left[\mathrm{S}\right]$ denotes the (time dependent) signal-molecule concentration, k_1, k_2 are the binding and dissociation rate constant, respectively, and the Hill coefficient indicates the number of signal molecules that must bind to the receptor. The signal-molecule concentration is controlled externally by the signal encoder and is not affected by reactions with the receptor as its concentration is assumed to be very low (as would be the case for binding to DNA). For pulsed signaling, we assume that the signal-molecule concentration takes on the square-wave oscillation

$$\left[\mathrm{S}\right] = \begin{cases} A, & (i-1)T \ \le t < (i-1)T + \Delta \\ 0, & (i-1)T + \Delta \le t < iT \end{cases} \qquad i = 1,2,..... \ , \tag{1}$$

as illustrated in Figure 2, where A is the pulse amplitude, T is the pulse period, Δ is the pulse duration, and $\gamma = \Delta / T$ is the duty cycle.

For sustained signaling, $\gamma = 1$ and the signal-molecule concentration is taken to jump from zero at t = 0 to a constant value of $\left[\mathrm{S}\right] = A$. Equation (5) is then easily solved to yield the time evolution of the binding probability for t > 0:

$$P_{sus}(t) = \bar{P}_{sus}\left[1 - e^{-t(k_1 A^n + k_2)}\right] \tag{S1}$$

where $\bar{P}_{sus}$ is the steady state probability at long times,

$$\bar{P}_{sus} = \lim_{t\to\infty} \ P_{sus}(t) = \frac{A^n}{K_A^n + A^n} \tag{S2}$$

and $K_A = (k_2 / k_1)^{1/n}$ (Eqn. 3) is the dissociation constant of RS_n.

For pulsed signaling, analytical solutions are known for square-wave signaling[2] and for sinusoidal signaling[3]) that include also adaptions for downstream product production and delays[4,5]. For square-wave signaling, the results are best represented by considering changes in binding that occurs during each individual period of the signaling. Introducing $\xi_i = t-(i-1)T$ as the time elapsed since the beginning of the *i*-th pulse, the binding change during this pulse can be expressed as a rising component appropriate for the time interval $0 \le \xi_i < \Delta$ during which the signal-molecule concentration is high,

$$\frac{P_i(\xi_i)}{\bar{P}_{sus}} = 1 - \frac{e^{-\xi_i(k_1A^n+k_2)}}{1-e^{-(k_1A^n\Delta+k_2T)}}\left[(1-e^{k_2\Delta-k_2T}) + e^{-i(k_1A^n\Delta+k_2T)}(e^{(k_1A^n+k_2)\Delta}-1)\right] \tag{6}$$

and a falling component for $\Delta \le \xi_i < T$ during which the signal-molecule concentration is low,

$$\frac{P_i(\xi_i)}{\bar{P}_{sus}} = \frac{(e^{k_2\Delta} - e^{-k_1A^n\Delta})(1-e^{-i(k_1A^n\Delta+k_2T)})}{1-e^{-(k_1A^n\Delta+k_2T)}} e^{-k_2\xi_i} \tag{7}$$

Figure 3 shows numerical values from these equations presented over a wide parameter space for n = 2, with Supplementary Figure S1 showing analogous results for n = 4. In these figures, K_A and k_1 are taken as the primary variables; the values of k_2 across this parameter space for the n = 2 and n = 4 cases are shown in Supplementary Figure S2a and S2b, respectively, while $P(t)$ at a selection of values of K_A are show in Supplementary Figure S3. From Eqns. (6) and (7), the binding probability $\bar{P}_i$ averaged over the *i*-th period can be determined to be

$$\begin{aligned}\bar{P}_i &= \frac{1}{T}\int_0^T P(\xi)d\xi = \frac{\bar{P}_{sus}}{T}\left[\Delta + \frac{(1-e^{-k_2(T-\Delta)})(e^{-(k_1A^n+k_2)\Delta}-1)}{(1-e^{-(k_1A^n\Delta+k_2T)})(k_1A^n+k_2)}\right] \\ &- \frac{\bar{P}_{sus}(1-e^{-(k_1A^n+k_2)\Delta})(e^{(k_1A^n+k_2)\Delta}-1)e^{-i(k_1A^n\Delta+k_2T)}}{T(k_1A^n+k_2)(1-e^{-(k_1A^n\Delta+k_2T)})} \\ &+ \frac{\bar{P}_{sus}(e^{k_2\Delta}-e^{-k_1A^n\Delta})(e^{-k_2\Delta}-e^{-k_2T})(1-e^{-i(k_1A^n\Delta+k_2T)})}{k_2T(1-e^{-(k_1A^n\Delta+k_2T)})}.\end{aligned} \tag{S3}$$

At long times, the average value of the binding for pulsed signaling therefore becomes

$$\bar{P}_{pulsed} = \lim_{i\to\infty}\bar{P}_i = \bar{P}_{sus}\gamma + \bar{P}_{sus}\frac{\omega\sigma}{1+\sigma}\frac{(1-e^{-\gamma(1+\sigma)/\omega})(1-e^{-(1-\gamma)/\omega})}{1-e^{-(1+\gamma\sigma)/\omega}} \tag{S4}$$

where

$$\omega = \frac{1}{k_2T} \text{ and } \sigma = \frac{k_1A^n}{k_2} = \frac{A^n}{K_A^n} . \tag{S5}$$

For fast pulsing (Eqn. (9), the oscillation period is much less than the dissociation time constant so $\omega \to \infty$ and the probability of association at long times becomes simply

$$\bar{P}_{pulsed} = \frac{\sigma\gamma}{1+\sigma\gamma}. \tag{S6}$$

These equations are the same form as those deduced for calcium signaling [2].

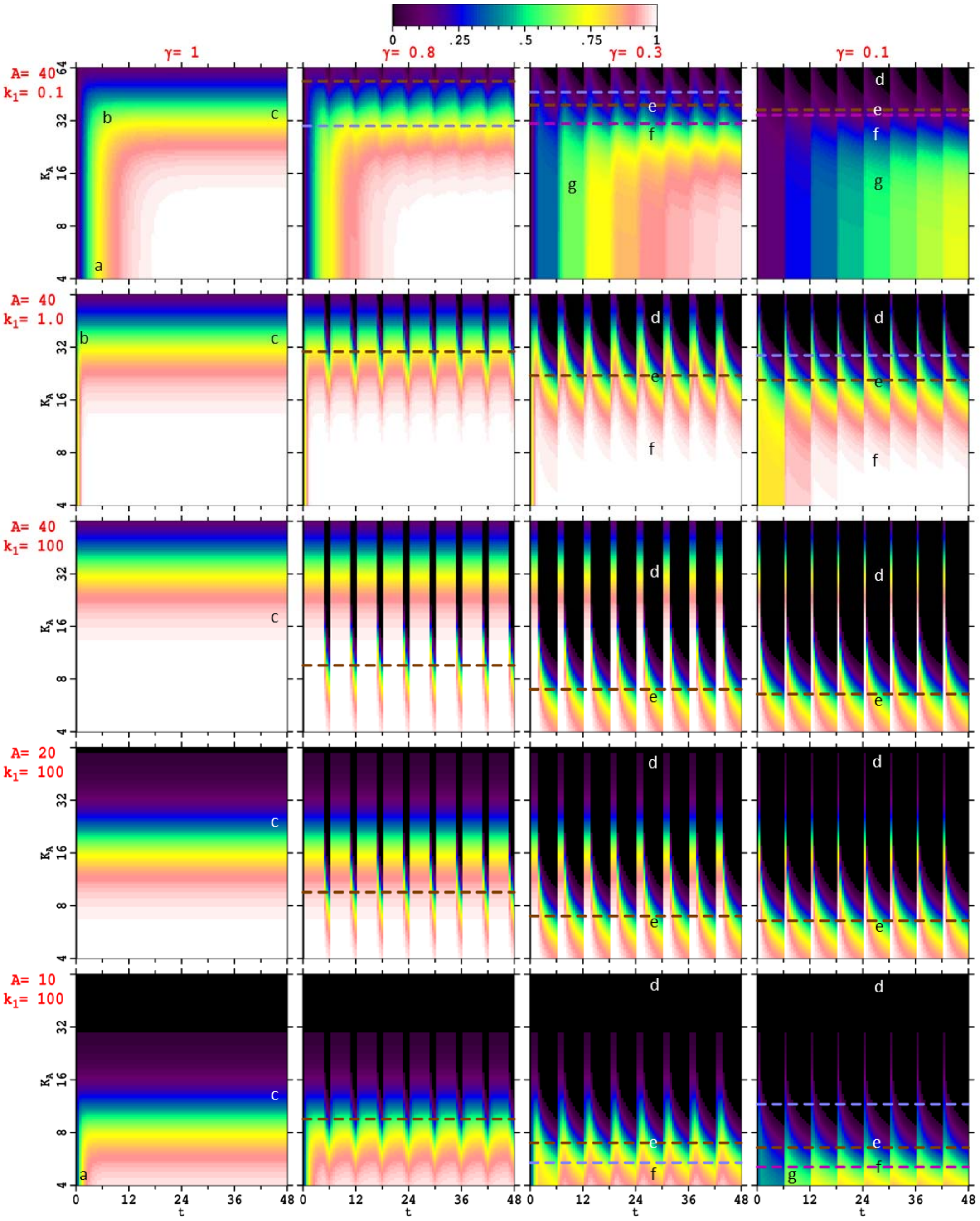

Supplementary Figure S1 | Promoter binding probabilities $P(t)$ (t in h) shown for n = 4 over the range of 4 – 64 nM in dissociation constants K_A, for various values of A (in nM) and k_1 (in 10^{-6} $nM^{-4}h^{-1}$), rows, and for various duty cycles γ (γ = 1 indicates sustained signaling), columns. The pulsing period is T = 6 h. The dashed lines indicate, when feasible, the maximum values of K_A satisfying inequalities Eqn. 9 (brown, fast pulsing limit), Eqn. 22 (grey, pulsing slows rise time), and Eqn. 24 (magenta, clocking such that binding increases with each pulse). Marked regimes are: *a*- *K*-independent initial binding, *b*- changeover, *c*-asymptotic regime, *d*- slow pulsing, *e*- competitive pulsing, *f*- fast pulsing, *g*- graded activation per pulse.

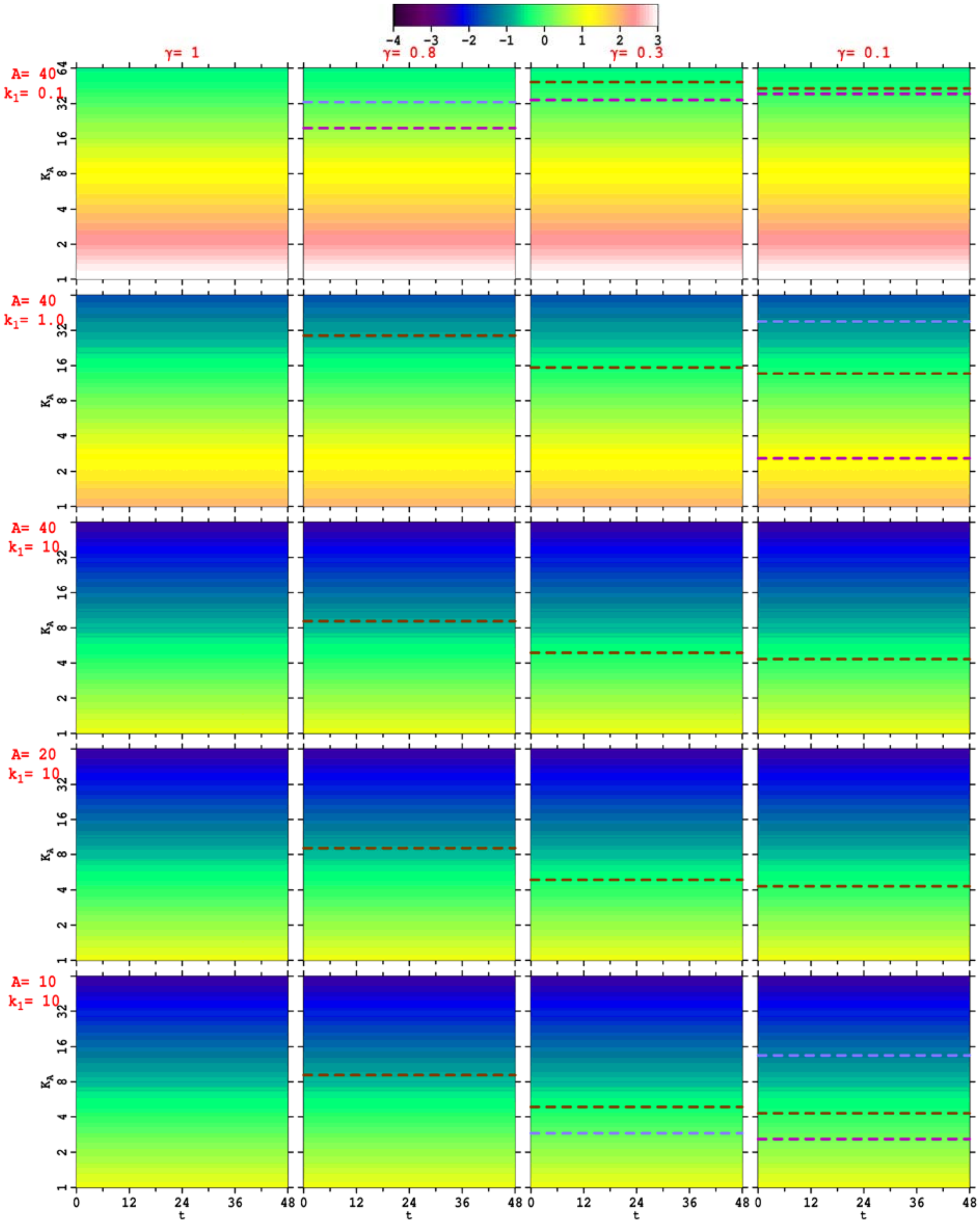

Supplementary Figure S2a. Values for $n = 2$ of k_2 over parameter space of Figure 3, where the scale shows $\log_{10}$ (k_2 / h) (i.e., there is a seven-order change in magnitude in k_2 from 10^{-4} h^{-1} (2.8×10^{-8} Hz) to 1000 h^{-1} (0.28 Hz). A range of 1 – 64 nM in dissociation constants K_A is shown for various values of A (in nM) and k_1 (in 10^{-3} $nM^{-2}h^{-1}$), with the pulsing period taken to be $T = 6$ h.

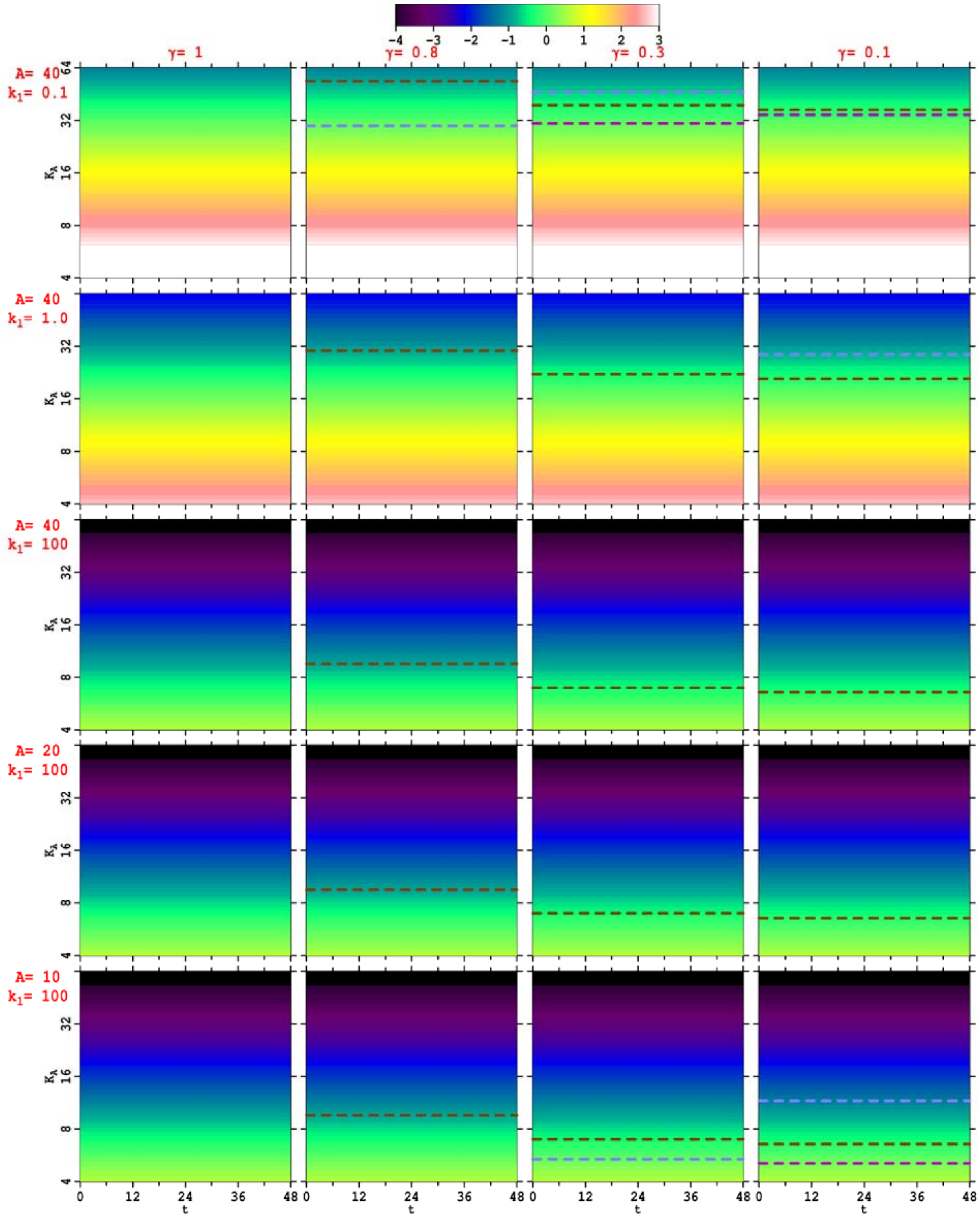

Supplementary Figure S2b. Values for n = 4 of k_2 over parameter space of Supplementary Figure S1, where the scale shows $\log_{10}$ (k_2 / h) (i.e., there is a seven-order change in magnitude in k_2 from 10^{-4} h^{-1} (2.8×10^{-8} Hz) to 1000 h^{-1} (0.28 Hz). A range of 4 – 64 nM in dissociation constants K_A is shown for various values of A (in nM) and k_1 (in 10^{-6} $nM^{-4}h^{-1}$), with the pulsing period taken to be T = 6 h.

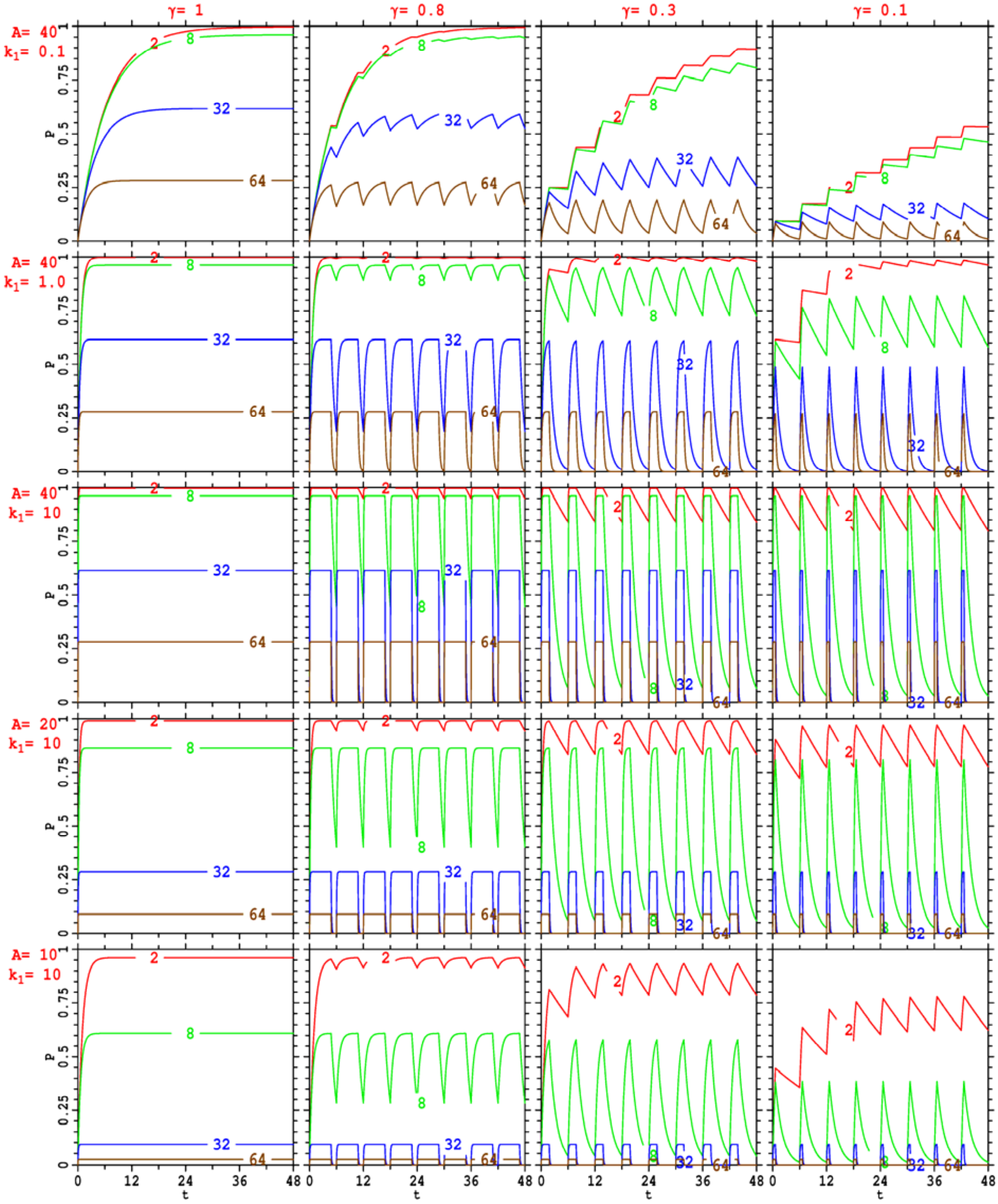

Supplementary Figure S3a. Extracts from Figure 3 showing for $n = 2$ individual dynamics $P(t)$ (t in h) at specific values of K_A = 2, 8, 32, and 64 nM, for various values of A (in nM) and k_1 (in 10^{-3} $nM^{-3}h^{-1}$), with the pulsing period taken to be $T = 6$ h.

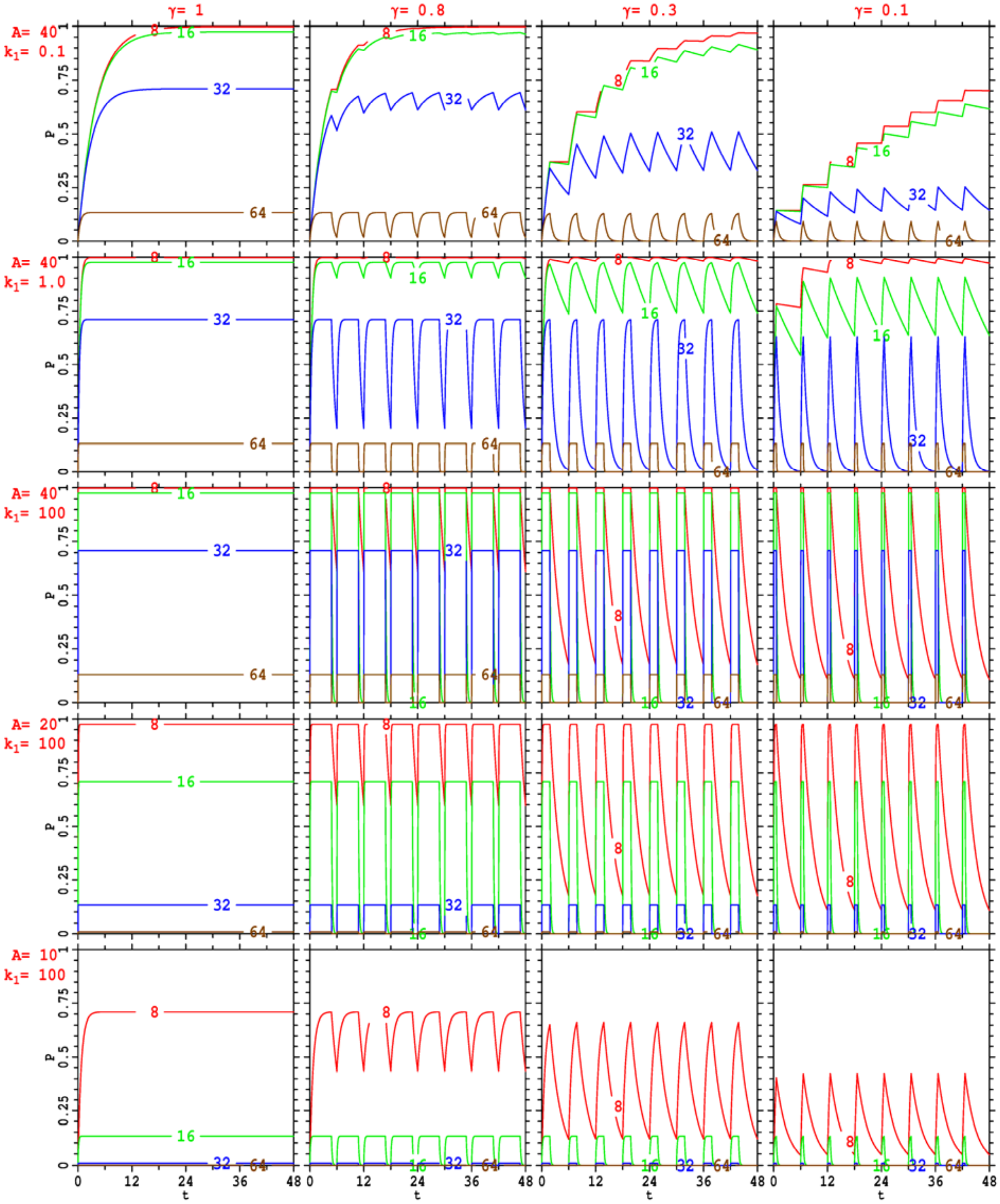

Supplementary Figure S3b. Extracts from Supplementary Figure S1 showing for n = 4 individual dynamics $P(t)$ (t in h) at specific values of K_A = 8, 16, 32, and 64 nM, for various values of A (in nM) and k_1 (in 10^{-6} $nM^{-4}h^{-1}$), with the pulsing period taken to be T = 6 h.

S4. The relationships between the steady mean binding probability, the average signal-molecule concentration, and the duty cycle

From. Eqn. (1), the average signal-molecule concentration is

$$\overline{[\mathrm{S}]} = \frac{1}{T}\int_{(i-1)T}^{iT}[\mathrm{S}]\,dt = A\gamma \,. \tag{15}$$

Using this relationship and Eqn. (S5), Eqn. (S6) can be rewritten to express the average binding probability as

$$\overline{P}_{pulsed} = \frac{\gamma A^n}{K_A^n + \gamma A^n}, \tag{14}$$

$$= \frac{\overline{[\mathrm{S}]}^n}{\gamma^{n-1}K_A^n + \overline{[\mathrm{S}]}^n} = \frac{\overline{[\mathrm{S}]}^n}{K_A'^n + \overline{[\mathrm{S}]}^n}$$

where

$$K_A' = \gamma^{(n-1)/n}K_A \tag{16}$$

is the apparent dissociation constant for pulsed signaling. Equation (14) shows that the average binding for pulsed signaling is reduced from that for signaling sustained at signal-molecule concentrations equal to the maximum amplitude A of the pulsed signal. However, this binding is much more than that obtained using signaling sustained at the average signal molecule concentration in the pulsed experiments. This occurs because n signal molecules must bind in order to activate gene expression, making periods of relatively high concentration very much more effective if $n > 1$. When $n = 4$, this non-linear effect becomes highly pronounced. An illustrative example of the principle can be seen by taking in arbitrary units $A = 1$, $K = 0.01$, and $\gamma = 0.1$. First, we consider pulsed signaling which, from Eqn. (14), leads to $\overline{P}_{pulsed} = 0.9$. Then we consider sustained signaling at either the peak signal-molecule concentration $A = 1$, or else the average signal-molecule concentration $\gamma A = 0.1$: the peak concentration yields $\overline{P}_{sus} = 1.0$ whereas the average concentration yields $\overline{P}_{sus} = 0.01$. Hence pulsed signaling increases the average binding hundredfold compared to sustained signaling at the low average signal-molecule concentration. However, if the same calculations are repeated using $n = 1$, then Eqn. (14) indicates that for pulsed signaling $\overline{P} = 0.01$, the same as for sustained signaling at the average signal-molecule concentration. These results are captured in Eqn. (16) which tells that pulsed binding reduces the apparent dissociation constant by the factor $\gamma^{(n-1)/n}$.

Another way of considering these results is through consideration of $\overline{P}_{pulsed}$ for changes in $\overline{\mathrm{S}}$:

$$\frac{d\overline{P}_{pulsed}}{d\overline{[\mathrm{S}]}} = \frac{n\overline{[\mathrm{S}]}^{n-1}\gamma^{n-1}K_A^n}{(\gamma^{n-1}K_A^n + \overline{[\mathrm{S}]}^n)^2} \,. \tag{S7}$$

Similarly for fast pulsing we have

$$\frac{d\overline{P}_{sus}}{d\overline{[\mathrm{S}]}} = \frac{n\overline{[\mathrm{S}]}^{n-1}K_A^n}{(K_A^n + \overline{[\mathrm{S}]}^n)^2} \tag{S8}$$

so that

$$\frac{d(\overline{P}_{pulsed}-\overline{P}_{sus})}{d\overline{[\mathrm{S}]}}=\frac{n\overline{[\mathrm{S}]}^{n-1}K_A^n(1-\gamma^{n-1})(\gamma^{n-1}K_A^{2n}-\overline{[\mathrm{S}]}^{2n})}{(\gamma^{n-1}K_A^n+\overline{[\mathrm{S}]}^n)^2(K_A^n+\overline{[\mathrm{S}]}^n)^2}\ . \qquad (\mathrm{S9})$$

Therefore,

$$\frac{d\overline{P}_{pulsed}}{d\overline{[\mathrm{S}]}}>\frac{d\overline{P}_{sus}}{d\overline{[\mathrm{S}]}}\ \text{ for }\ \overline{[\mathrm{S}]}<\gamma^{1/2n}K_A\ , \qquad (\mathrm{S10})$$

which shows signal pulses enhance binding with the receptor at low signal-molecule levels. A similar mechanism was found that calcium oscillations increase calcium sensitivity of gene transcription at low levels of stimulation[6].

S5. Times to reach steady-state binding driven by pulsed and sustained signaling

For sustained signaling, the relaxation time to reach the steady binding probability is given simply from Eqn. (S1) as

$$\tau_{sus}=\frac{1}{k_1A^n+k_2}=\frac{1}{k_1(A^n+K_A^n)} \qquad (19)$$

For pulsed signaling, the relaxation time to attain the steady mean binding probability is defined as

$$\tau_{pulsed}=\frac{T\sum_{i=1}^{\infty}(\overline{P}-\overline{P_i})}{\overline{P}}\ . \qquad (\mathrm{S11})$$

From Eqns. (S2) and (6)-(7), we have

$$\tau_{pulsed}=\frac{Te^{-(k_1A^n+k_2T)}}{1-e^{-(k_1A^n+k_2T)}}\times \left[\frac{\frac{k_2}{k_1A^n+k_2}(1-e^{-(k_1A^n+k_2)\Delta})(e^{(k_1A^n+k_2)\Delta}-1)+(e^{k_2\Delta}-e^{-k_1A^n\Delta})(e^{-k_2\Delta}-e^{-k_2T})}{k_2\Delta(1-e^{-(k_1A^n\Delta+k_2T)})+\frac{k_1A^n}{k_1A^n+k_2}(1-e^{-k_2(T-\Delta)})(1-e^{-(k_1A^n+k_2)\Delta})}\right], \qquad (\mathrm{S12})$$

which can be expanded in a Taylor expansion in the fast-pulsing limit $k_2T\to 0$ for fixed duty cycle γ to give[2]

$$\tau_{pulsed}=\frac{1}{k_1(A^n\gamma+K_A^n)}-\frac{T}{2}(1-\gamma)+O(T^2)\ . \qquad (\mathrm{S13})$$

Comparing Eqns. (6) and (7), it is clear that $\tau_{pulsed} \rightarrow \tau_{sus}$ as $\gamma \rightarrow 1$, but ignoring the higher order terms in Eqn. (S12), steady state is achieved much slower ($\tau_{pulsed} > \tau_{sus}$) for pulsed signaling when the pulse frequency is high, i.e., when

$$T < \frac{2k_1 A^n}{(k_1 A^n + k_2)(k_1 A^n \gamma + k_2)} \ . \tag{21}$$

This period may be less than or greater than that required for the fast-pulsing approximation used in the above derivation, so always the most stringent condition needs to be considered.

S6. The relationship between binding probability and number of pulses during the initial stages of relaxation

From Eqn. (6), the ratio of the binding probability at the end of the *i*-th pulse to that at the end of the previous pulse is given by

$$\frac{P_i(T)}{P_{i-1}(T)} = \frac{1 - e^{-i(k_1 A^n \Delta + k_2 T)}}{1 - e^{-(i-1)(k_1 A^n \Delta + k_2 T)}} \ . \tag{S14}$$

Setting

$$\alpha = e^{-(k_1 A^n \Delta + k_2 T)} = e^{-(k_1 A^n \gamma + k_2)T} \tag{S15}$$

this simplifies to

$$P_i(T) = \frac{1-\alpha^i}{1-\alpha} P_1(T) \tag{S16}$$

where, from Eqn. (7),

$$P_1(T) = \bar{P}_{sus}\left[1 - e^{-(k_1 A^n + k_2)\gamma T}\right] e^{-k_2 T(1-\gamma)} = \bar{P}_{sus}\left[1 - e^{-k_1 \gamma (A^n + K_A^n)T}\right] e^{-k_1 K_A^n T(1-\gamma)} \tag{S17}$$

analogous to the equations describing Ca^{2+} signaling[7,8]. Let $\theta = 1-\alpha$, then from Eqn. (S15) $\theta \rightarrow 0$ whenever

$$K_A^n << \frac{1}{k_1 T} - \gamma A^n \ , \tag{24}$$

allowing a Taylor expression of Eqn. (S16) yielding

$$P_i(T) = \frac{1-(1-\theta)^i}{\theta} P_1(T) \approx i P_1(T) \, . \tag{S18}$$

Hence the binding probability under the *i*-th pulse steadily increases whenever Eqn. (24) holds. Using Eqn. (3), this condition can also be written as

$$k_2 T << 1 - k_1 A^n \gamma T \tag{S19}$$

and so is stricter than the limit for fast pulsing, Eqn. (9).

S7. Cumulative signal molecule exposure and cumulative binding

There is much interest in the "cumulative signal" c^{S} and associated "cumulative binding probability" c^{P} associated with pulsed signaling. Cumulative signal is defined as the total time exposure of the system to signal molecules after reaction time t':

$$c^{\mathrm{S}}(t') = \int_0^{t'} [\mathrm{S}]dt \tag{S20}$$

while

$$c^{\mathrm{P}}(t') = \int_0^{t'} P(t)dt\,. \tag{S21}$$

gives the cumulative binding. Of concern is the ratio of cumulative binding from sustained signaling to that from pulsed signaling given the same amount of cumulative signal exposure in each case. This involves comparing the cumulative binding at different times t' for sustained signaling and t'' for pulsed signaling that produce the same cumulative signal-molecule level, which from Eqns. (5) and (S20) is

$$\begin{aligned} c^{\mathrm{S}} \equiv c^{\mathrm{S}}_{sus}(t') &= \int_0^{t'} Adt = At' \\ = c^{\mathrm{S}}_{pulsed}(t'') &= \int_0^{t''} \gamma Adt = \gamma At'' \end{aligned} \tag{S22}$$

at the end of each pulse. The solution of this equation is simply

$$t' = \gamma t'', \tag{S23}$$

which states nothing more than the relative rates at which pulsed and sustained signaling delivers the signal molecule. The ratio of the cumulative bindings therefore becomes

$$\frac{c^{\mathrm{P}}_{sus}(c^{\mathrm{S}})}{c^{\mathrm{P}}_{pulsed}(c^{\mathrm{S}})} = \frac{\int_0^{\gamma t''} P_{sus}(t)dt}{\int_0^{t''} P_{pulsed}(t)dt}\,. \tag{S24}$$

In the asymptotic limit of $c^{\mathrm{S}} \to \infty,\quad t'' \to \infty$ and therefore

$$\frac{c^{\mathrm{P}}_{sus}(c^{\mathrm{S}})}{c^{\mathrm{P}}_{pulsed}(c^{\mathrm{S}})} \to \frac{\gamma t'' \bar{P}_{sus}}{t'' \bar{P}_{pulsed}} = \gamma \frac{\bar{P}_{sus}}{\bar{P}_{pulsed}}\,. \tag{S25}$$

From Eqns. (12) and (13), this evaluates to

$$\lim_{c^s \to \infty} \frac{c^P_{sus}(c^S)}{c^P_{pulsed}(c^S)} = \begin{cases} 1 & \text{for slow pulsing} \\ \dfrac{K_A^n + \gamma A^n}{K_A^n + A^n} & \text{for fast pulsing} \end{cases} \tag{18}$$

This result for fast pulsing approaches γ for high-affinity reactions and 1 for low-affinity reactions. Pulsing therefore always increases somewhat the cumulative binding at the same level of cumulative signal molecule. In Supplementary Figure S4, the ratio of the cumulative binding is shown as a function of the ratio of cumulative signal molecule from pulsed signaling to that manifested in the simulations at t'' = 48 h, its characteristic features defined by Eqn. (18).

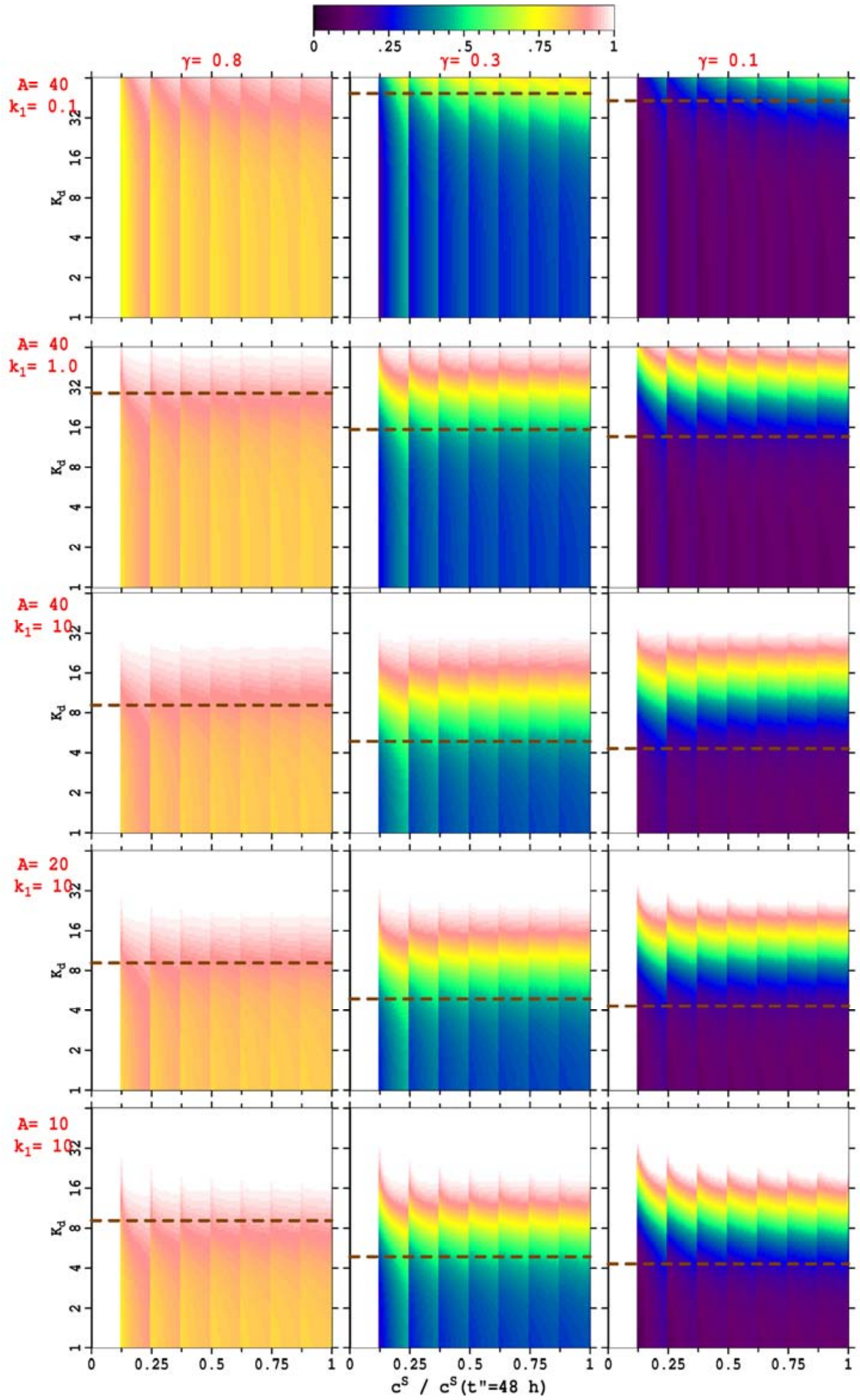

Supplementary Figure S4a. The ratio of the cumulative binding for n = 2 from sustained signaling to that from pulsed signaling is shown for the parameter space of Figure 3: a range of 1 – 64 nM in dissociation constants K_A, for various values of A (in nM) and k_1 (in 10^{-3} $nM^{-2}h^{-1}$), with the pulsing period taken to be T = 6 h. The brown dashed lines indicate the crossover between fast and slow pulsing, Eqn. (9).

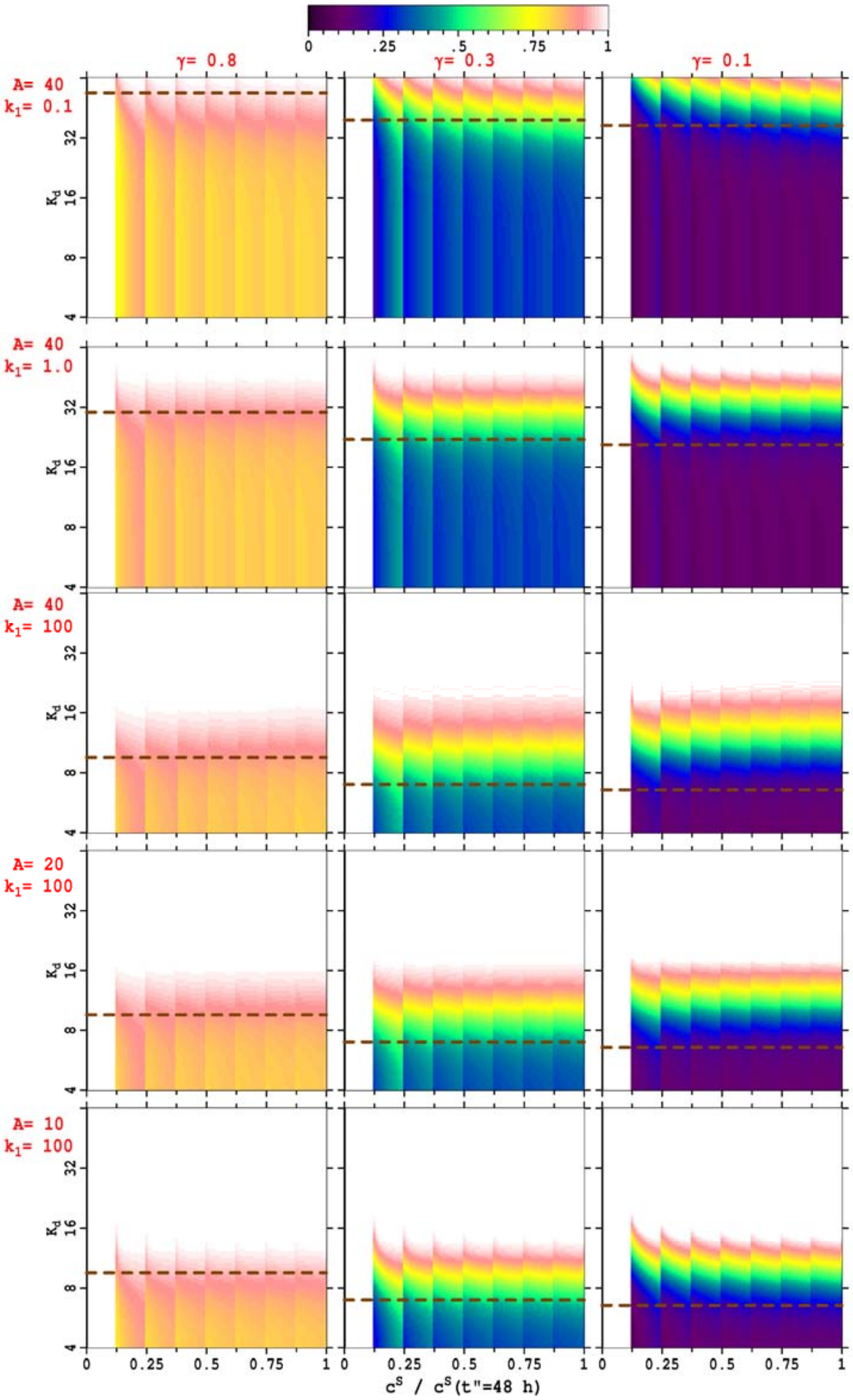

Supplementary Figure S4b. The ratio of the cumulative binding for n = 4 from sustained signaling to that from pulsed signaling is shown for the parameter space of Supplementary Figure S1: a range of 4 – 64 nM in dissociation constants K_A, for various values of A (in nM) and k_1 (in 10^{-6} $nM^{-4}h^{-1}$), with the pulsing period taken to be T = 6 h. The brown dashed lines indicate the crossover between fast and slow pulsing, Eqn. (9).

S8. Some other ways in which Hill-type signaling can manifest when 4 signal molecules must bind to the receptor a la p53 binding.

In the main text, the equations are presented showing how binding of p53 dimers to DNA can be interpreted using a simple Hill equation, focusing on the limiting situations of weak and strong dimerization. Alternatively, if all four p53 molecules pre-associate into a tetramer that binds as a single unit to DNA, then the reactions can be described most simply as

$$4\text{p53} \underset{k_{2t}}{\overset{k_{1t}}{\rightleftarrows}} \text{p53}_4 \qquad K_{Ad}^3 = \frac{k_{2t}}{k_{1t}} = \frac{[\text{p53}]^4}{[\text{p53}_2]} \tag{S26}$$

followed by receptor binding

$$\text{p53}_4 + \text{DNA} \underset{k_{2R}}{\overset{k_{1R}}{\rightleftarrows}} \text{DNA.4p53}, \qquad K_{AR} = \frac{k_{2R}}{k_{1R}} = \frac{[\text{p53}_4][\text{DNA}]}{[\text{DNA.p53}_4]} \ . \tag{S27}$$

It is possible to obtain analytic solutions to these coupled equations. By analogy to Eqn. (8), the asymptotic binding probability at $t = \infty$ for sustained signaling becomes

$$\bar{P}_{sus} = \frac{[\text{DNA.p53}_4]}{[\text{DNA}]_{\text{total}}} = \frac{[\text{p53}_4]}{K_{AR} + [\text{p53}_4]} \ . \tag{S28}$$

Ignoring the amount of p53 bound to the DNA, the mass balance equation accounting for the total p53 concentration $[\text{p53}]_{\text{total}}$ becomes

$$[\text{p53}]_{\text{total}} = [\text{p53}] + 4[\text{p53}_4] \ . \tag{S29}$$

Analytical solution of these equations leads to a fourth-order polynomial to solve which yields in the limits of strong and weak tetramerization

$$\begin{aligned} K_{At} \ll [\text{p53}]_{\text{total}}: \qquad & \bar{P}_{sus} = \frac{[\text{p53}]_{\text{total}}/4}{K_{AR} + [\text{p53}]_{\text{total}}/4}, \text{ and} \\ K_{At} \gg [\text{p53}]_{\text{total}}: \qquad & \bar{P}_{sus} = \frac{[\text{p53}]_{\text{total}}^4/K_{Ad}^3}{K_{AR} + [\text{p53}]_{\text{total}}^4/K_{Ad}^3}, \end{aligned} \tag{S30}$$

respectively, presenting effective Hill coefficients of 1 (strong tetramerization) and 4 (weak tetramerization). This equation directly parallels Eqn. (32) for dimer-of-dimer binding.

Another variation is the possibility that the binding of p53 to the DNA occurs molecule at a time without any pre-association. Many possibilities exist for how the rate constants and equilibrium constants change with each subsequent p53 addition. Consider the situation in which the first addition is significantly different to the subsequent three. The appropriate reactions are first

$$\text{p53} + \text{DNA} \underset{k_{21}}{\overset{k_{11}}{\rightleftarrows}} \text{DNA.p53} \qquad K_{A1} = \frac{k_{21}}{k_{11}} = \frac{[\text{p53}][\text{DNA}]}{[\text{DNA.p53}]} \tag{S31}$$

followed by the remaining processes

$$3\text{p53}+\text{DNA.p53}\underset{k_{2i}}{\overset{k_{1i}}{\rightleftarrows}}\text{DNA.4p53},\qquad K_{Ai}^3=\frac{k_{2i}}{k_{1i}}=\frac{[\text{p53}]^3[\text{DNA.p53}]}{\text{DNA.4p53}}\ . \tag{S32}$$

Ignoring the amount of p53 bound to the DNA, the p53 concentration is conserved at $[\text{p53}]_{\text{total}}$, whilst mass balance for the DNA yields

$$[\text{DNA}]_{\text{total}}=[\text{DNA}]+[\text{DNA.p53}]+[\text{DNA.4p53}]\ . \tag{S33}$$

These equations can be solved to give

$$\bar{P}_{sus}=\frac{[\text{DNA.4p53}]}{[\text{DNA}]_{\text{total}}}=\frac{[\text{p53}]_{\text{total}}^4}{K_{Ai}^3\left(K_{A1}+[\text{p53}]_{\text{total}}\right)+[\text{p53}]_{\text{total}}^4} \tag{S34}$$

which simplifies in the strong binding and weak binding limits to

$$K_{A1}\ll[\text{p53}]_{\text{total}}:\qquad \bar{P}_{sus}=\frac{[\text{p53}]_{\text{total}}^3}{K_{Ai}^3+[\text{p53}]_{\text{total}}^3},\ \text{and}$$

$$K_{A1}\gg[\text{p53}]_{\text{total}}:\qquad \bar{P}_{sus}=\frac{[\text{p53}]_{\text{total}}^4}{K_{A1}K_{Ai}^3+[\text{p53}]_{\text{total}}^4}, \tag{S35}$$

respectively. In the strong binding limit, the first p53 binds immediately but inhibits subsequent binding, giving a Hill coefficient of 3, whereas in the weak binding limit the first p53 is difficult to bind but then the remaining three bind cooperatively, generating a Hill coefficient of 4.

Expanding on this solution, the sequential binding process can be represented generally as

$$\begin{aligned}
&\text{p53}+\text{DNA}\underset{k_{21}}{\overset{k_{11}}{\rightleftarrows}}\text{DNA.p53} && K_{A1}=\frac{k_{21}}{k_{11}}=\frac{[\text{p53}][\text{DNA}]}{[\text{DNA.p53}]},\\
&\text{p53}+\text{DNA.p53}\underset{k_{22}}{\overset{k_{12}}{\rightleftarrows}}\text{DNA.2p53}, && K_{A2}=\frac{k_{22}}{k_{12}}=\frac{[\text{p53}][\text{DNA.p53}]}{[\text{DNA.2p53}]},\\
&\text{p53}+\text{DNA.2p53}\underset{k_{23}}{\overset{k_{13}}{\rightleftarrows}}\text{DNA.3p53}, && K_{A3}=\frac{k_{23}}{k_{13}}=\frac{[\text{p53}][\text{DNA.2p53}]}{[\text{DNA.3p53}]},\ \text{and}\\
&\text{p53}+\text{DNA.3p53}\underset{k_{24}}{\overset{k_{14}}{\rightleftarrows}}\text{DNA.4p53}, && K_{A4}=\frac{k_{24}}{k_{14}}=\frac{[\text{p53}][\text{DNA.3p53}]}{[\text{DNA.4p53}]}
\end{aligned} \tag{S36}$$

for which the asymptotic solution is

$$\bar{P}_{sus}=\frac{[\text{p53}]_{\text{total}}^4}{K_{A1}K_{A2}K_{A3}K_{A4}+K_{A2}K_{A3}K_{A4}[\text{p53}]_{\text{total}}+K_{A3}K_{A4}[\text{p53}]_{\text{total}}^2+K_{A4}[\text{p53}]_{\text{total}}^3+[\text{p53}]_{\text{total}}^4} \tag{S37}$$

Hence depending on the dissociation constants a range of scenarios with Hill coefficients between 1 and 4 can emerge. Of particular note is the cooperative binding scenario in which the first reaction is of low affinity but the subsequent ones have high affinity such that

$$\begin{aligned} &K_{A4} << [\mathrm{p53}]_{\mathrm{total}} \\ &K_{A3}K_{A4} << [\mathrm{p53}]_{\mathrm{total}}^{2} \\ &K_{A2}K_{A3}K_{A4} << [\mathrm{p53}]_{\mathrm{total}}^{3} \\ &K_{A1}K_{A2}K_{A3}\,\mathrm{K}_{A4} \sim [\mathrm{p53}]_{\mathrm{total}}^{4} \end{aligned} \tag{S38}$$

then the simplistic scenario

$$\bar{P}_{sus} = \frac{[\mathrm{p53}]_{\mathrm{total}}^{4}}{K_{A1}K_{A2}K_{A3}K_{A4} + [\mathrm{p53}]_{\mathrm{total}}^{4}} \tag{S39}$$

emerges.

S9. Supplementary References

1 Weinberg, R. L., Veprintsev, D. B., Bycroft, M. & Fersht, A. R. Comparative Binding of p53 to its Promoter and DNA Recognition Elements. *J. Mol. Biol.* **348**, 589-596 (2005).

2 Salazar, C., Politi, A. Z. & Höfer, T. Decoding of Calcium Oscillations by Phosphorylation Cycles: Analytic Results. *Biophys. J.* **94**, 1203-1215 (2008).

3 Wee, K. B., Yio, W. K., Surana, U. & Chiam, K. H. Transcription factor oscillations induce differential gene expressions. *Biophys. J.* **102**, 2413-2423 (2012).

4 Tiana, G., Jensen, M. H. & Sneppen, K. Time delay as a key to apoptosis induction in the p53 network. *European Physical Journal B* **29**, 135-140 (2002).

5 Liu, B., Yan, S. & Wang, Q. Delay Hill dynamics in regulatory biological systems. *Molecular BioSystems* **7**, 457-463 (2011).

6 Dolmetsch, R. E., Xu, K. & Lewis, R. S. Calcium oscillations increase the efficiency and specificity of gene expression. *Nature* **392**, 933-936 (1998).

7 Keener, J. & Sneyd, J. *Mathematical Physiology*. (Springer-Verlag, New York, 1998).

8 Bertram, R., Sherman, A. & Stanley, E. F. Single-domain/bound calcium hypothesis of transmitter release and facilitation. *J. Neurophysiol.* **75**, 1919-1931 (1996).
